# Hypothermic temperature effects on organ survival and restoration

**DOI:** 10.1038/srep09563

**Published:** 2015-04-22

**Authors:** Jun Ishikawa, Masamitsu Oshima, Fumitaka Iwasaki, Ryoji Suzuki, Joonhong Park, Kazuhisa Nakao, Yuki Matsuzawa-Adachi, Taro Mizutsuki, Ayaka Kobayashi, Yuta Abe, Eiji Kobayashi, Katsunari Tezuka, Takashi Tsuji

**Affiliations:** 1 Department of Biological Science and Technology, Graduate School of Industrial Science and Technology, Tokyo University of Science, Noda, Chiba. 278-8510, JAPAN; 2 Laboratory for Organ Regeneration, RIKEN Center for Developmental Biology, Kobe, Hyogo. 650-0047, JAPAN; 3 Research Institute for Science and Technology, Tokyo University of Science, Noda, Chiba. 278-8510, JAPAN; 4 Department of Surgery, Keio University, School of Medicine, Shinanomachi, Shinjuku-ku, Tokyo, 160-8582, JAPAN; 5 Department of Organ Fabrication, Keio University, School of Medicine, Shinanomachi, Shinjuku-ku, Tokyo 160-8582, JAPAN; 6 Center for Development of Advanced Medical Technology, Jichi Medical University, Shimotsuke, Tochigi. 329-0431, JAPAN; 7 Organ Technologies Inc., Tokyo. 101-0048, JAPAN

## Abstract

A three-dimensional multicellular organism maintains the biological functions of life support by using the blood circulation to transport oxygen and nutrients and to regulate body temperature for intracellular enzymatic reactions. Donor organ transplantation using low-temperature storage is used as the fundamental treatment for dysfunctional organs. However, this approach has a serious problem in that donor organs maintain healthy conditions only during short-term storage. In this study, we developed a novel liver perfusion culture system based on biological metabolism that can maintain physiological functions, including albumin synthesis, bile secretion and urea production. This system also allows for the resurrection of a severely ischaemic liver. This study represents a significant advance for the development of an *ex vivo* organ perfusion system based on biological metabolism. It can be used not only to address donor organ shortages but also as the basis of future regenerative organ replacement therapy.

Multicellular organisms are composed of various organs and tissues comprising specialised functional cells that have a precise three-dimensional (3D) arrangement in the living body^1^. The organ systems include the nervous system, digestive system and circulatory system, and they are essential for maintaining fully functional networks in a living body. The nervous system, with its sympathetic and parasympathetic nerves, antagonistically regulates organ functions such as the heartbeat, gastrointestinal motility and thermoregulation^2^. To maintain homeostasis, the digestive system plays crucial roles in the digestion and absorption of various nutrients^3^. Multicellular organisms can be supported by oxygen immobilised in erythrocytes, and by nutrients, hormones and biological materials solubilised in the blood serum through the circulatory system, including the heart, lungs and sterical vascular network^4^. The materials that are transported through the organ network systems are essential for cell proliferation and the physiological functions of various cells.

Vascular networks, comprising arteriovenous vessels and microvessels, contribute to 3D tissue formation by supplying blood containing oxygen and nutrients^5^. Blood contains various cell types, including erythrocytes and immune-competent cells for immunological defence, and these cells are involved in life support and homeostasis. Blood serum plays essential roles in various biological functions, including the transportation of nutrients and bioactive factors and the transitional regulation of colloidal osmotic pressure by serum proteins^6^. The blood and vessels are responsible for heat dissipation via vasoconstriction and vasodilatation in peripheral regions such as the fingers, palms and ear lobes, and they also contribute to thermal regulation, which strongly affects cellular proliferation and functions^7^,^8^. Decreased body temperature, or hypothermia, causes depression of the heartbeat and respiration, and life ends due to the degradation of various metabolic factors^9^. Therapeutic hypothermia, which induces metabolic suppression in response to temperatures between 30 and 33°C, has been used to treat patients experiencing subarachnoid haemorrhage and cerebral infarction^10^. Severe hypothermia, which occurs at temperatures between 20 and 28°C, causes a reduction of physiological functions such as heart rate, respiratory rate and blood pressure^11^. However, hypothermia below a body temperature of 20°C is lethal. In severe and lethal hypothermia, reduced levels of adenosine triphosphate (ATP), which is generated by glycolysis and plays an essential role in the support of cell activities, have been reported^12^. However, the fundamental factors that regulate the clinical outcomes of severe and lethal hypothermia have not yet been clarified.

Organ transplantation is currently used to replace a dysfunctional organ and to restore organ function *in vivo*^13^. The donor organs are stored using static cold preservation systems using various organ preservation solutions including intracellular- or extracellular-type fluids during organ transportation and pre-treatment of the recipient^14^. Although these organ preservation methods have been developed, there is a worldwide donor organ shortage that remains an unresolved problem^15^. To effectively use organs from the limited number of donors, new approaches are being developed to prolong the organ preservation time by storing organs at sub-zero temperatures^43^. Techniques are currently being developed to increase the number of donor organs by expanding the use of organs from extended criteria donors (ECDs) and donation after cardiac death (DCD)^16^. Thus, the development of organ preservation methods and resuscitation methods for successful and safe transplantation of marginal donor organs such as ECDs and DCDs are greatly needed. Recently, various preservation methods using machine perfusion systems combined with organ preservation solutions, which are modified using culture medium^17^ and blood components^18^ under cold or normothermic temperatures, have also been developed to decrease the risk of serious post-operative complications associated with graft survival and dysfunction^14^,^19^,^20^. However, physiological activities of the organ were strongly depressed under static cold storage, and various metabolites that are necessary for cellular activities, such as amino acids, proteins and ATP, were deficient in the preserved organ. Additional novel concepts and technological developments for organ preservation based on biological responses and mechanisms have also been expected for donor expansion in organ transplantation therapy^15^,^21^.

In this study, we demonstrate the essential factors, including oxygen supply using erythrocytes and hypothermic regulation at 22°C, necessary for the survival and restoration of organ functions. Our tests are based on biological responses such as cell activity, cellular disorders and metabolism using our developed 3D liver perfusion culture system. In a metabolome analysis using cultured hepatocytes, we determined that a biological window for cell survival, but not cell proliferation and function, exists in the hypothermic zone of approximately 22°C, and we found that hypothermic temperature could regulate the biological factors involved in cell survival, such as ATP synthesis, amino acid synthesis and glucose metabolism. This 3D perfusion culture based on biological responses was able to preserve a donor liver *in vitro* and could be used to resuscitate a DCD liver. It also has the potential to replace donor organ transplantation and regeneration *in vivo*. This study thus represents a concept that has considerable potential as a novel organ preservation and restoration method for the next generation of 3D organ culture.

## Results

### Optimisation of a 3D liver perfusion culture system using erythrocytes at hypothermic temperature

**Figure 1:**
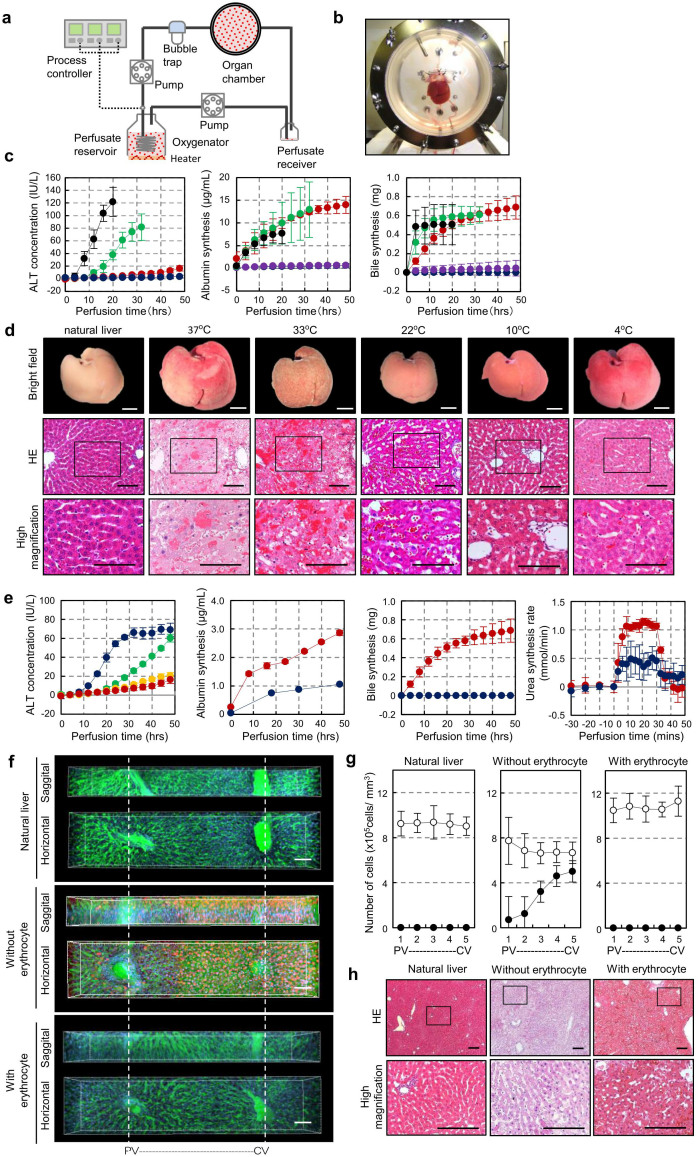
Establishment and optimisation of the liver perfusion culture system. (a), Diagram of the *ex vivo* organ perfusion culture circuit. (b), Photograph of isolated liver placement by our hanging method in the organ chamber. (c), Assessments of ALT activity (*left*), albumin synthesis (*centre*) and bile production (*right*) during liver perfusion culture at 22°C with 5.0 × 10^11^ cells/L erythrocytes. The coloured lines represent temperatures of 37°C (black), 33°C (green), 22°C (red), 10°C (purple) and 4°C (blue). (d), Photographs (*top*) and histological images (*middle and bottom*) of the cultured livers at different temperatures. Higher-magnification images are shown in the boxed area (*bottom*). Scale bars, 100 μm. (e), Assessments of ALT activity (*left*), albumin synthesis (*centre left*), bile production (*centre right*) and urea synthesis (*right*) during liver perfusion culture at 22°C with/without erythrocytes. These data represent erythrocyte concentrations of 0.5 × 10^11^ cells/L (green), 2.0 × 10^11^ cells/L (orange), with 5.0 × 10^11^ cells/L (red) and no erythrocytes (blue). (f), 3D images of sinusoidal structure in natural (*top column*) and cultured livers after 48 hr of perfusion at 22°C with/without erythrocyte (*middle column and bottom column*). The sagittal section (*top*) and horizontal section (*bottom*) are shown in a hepatic lobule between the central vein (CV) and the portal vein (PV). The vascular structure is represented by FITC (green), and dead cells (red) are indicated by the propidium iodide staining. All sections are counterstained with Hoechst 33342 (blue). Scale bars: 100 μm. (g), The numbers of total cells (white) and dead cells (black) in the natural (*left*) and cultured livers after 48 hrs at 22°C with/without erythrocytes (*centre and right*). These cells are counted in the optional areas evenly between CV and PV in a range of 100 μm (wide) × 200 μm (depth) × 100 μm (height). (h), Histological analysis of natural (*left*) and cultured livers after 48 hrs at 22°C with/without erythrocytes (*centre and right*). Higher-magnification images are shown in the boxed area (*bottom*). Scale bars, 100 μm.

To establish a 3D organ perfusion culture system, we designed an extracorporeal liver perfusion circuit that regulates the oxygen supply and culture temperature. The circuit was connected to the portal vein and hepatic upper inferior vena cava of an isolated rat liver (Fig. 1a, b). The isolated liver was placed in a culture device filled with culture medium to achieve sufficient perfusion flow and had similar intrahepatic hemodynamic to natural liver (Fig. 1b). This hanging method successfully maintained the flow of culture medium so that it was well perfused into all lobes through vascular networks of the cultured liver. Such perfusion of the cultured liver was not achievable with the putting liver as represented by the angiogram image and trypan blue infusion data shown in Supplementary Fig. 1a. We first examined how the culture temperature affected the physiological functions of the liver cultured in this system. At temperatures of 4, 10, 22, 33 and 37°C, we evaluated the concentration of alanine aminotransferase (ALT) as a hepatic disorder marker, and we measured albumin and bile as variables representing liver-specific physiological functions. After perfusion at 37°C for 20 hours (hrs) and 33°C for 32 hrs, ALT concentrations of 121 IU/L and 81 IU/L were observed, respectively. By contrast, the ALT concentrations of the perfusion cultures at 4, 10 and 22°C remained low (Fig. 1c, *left*). Continuous high-level albumin and bile synthesis was observed only in the culture at 22°C after 48 hrs, not in cultures at 4 and 10°C (Fig. 1c, *centre* and *right*). Swelling and disorganisation of the sinusoidal structure were observed in the cultures at 37 and 33°C after 48 hrs. By contrast, the sinusoidal structures were successfully maintained and were equivalent to those in a living liver in the livers cultured at 4, 10 and 22°C (Fig. 1d).

We next used erythrocytes to analyse the effect of oxygen supply on liver damage and physiological functions. With the perfusion culture using L-15 medium containing 10% FCS with a dissolved oxygen concentration at 6.77 ppm without erythrocytes, the ALT concentration drastically increased after 10 hrs (Fig. 1e). By contrast, ALT release was supressed by the addition of erythrocytes in a dose-dependent manner, and the cultured liver with erythrocytes at a density of 5 × 10^11^ cells/L was sufficiently maintained after 48 hrs of culture in terms of not only the ALT concentration but also the physiological functions, such as the changing of the pH of the medium, albumin and bile secretion, and urea synthesis rate (Fig. 1e and Supplementary Fig. 1b). Histological analysis also revealed the effects of the erythrocytes in the 22°C culture condition on the maintenance of the cultured liver (Supplementary Fig. 1c). To further evaluate the preservative effect of erythrocytes in the 22°C culture condition, we performed 3D image analysis, in which both the structural maintenance of the sinusoidal network and hepatocyte survival could be represented using a fluorescein 5-isothiocyanate (FITC)-conjugated gelatine and propidium iodide (PI) in a hepatic lobule (Fig. 1f). The PI-positive dead hepatocytes (red to orange) and the destruction of the sinusoidal network by leaking of FITC conjugated gelatine were clearly observed around the central vein but not the hepatic portal vein in the culture without erythrocytes (Fig. 1f, g, h). By contrast, the sinusoidal network and hepatocyte survival of the cultured liver cultured with erythrocytes were successfully maintained at equivalent states to those of the natural liver (Fig. 1f, g, h). These findings indicate that our perfusion culture system optimised the use of erythrocytes and that the 22°C culture condition shows potential for long-term organ preservation.

### Evaluation of cellular physiological activity due to culture temperature change

**Figure 2:**
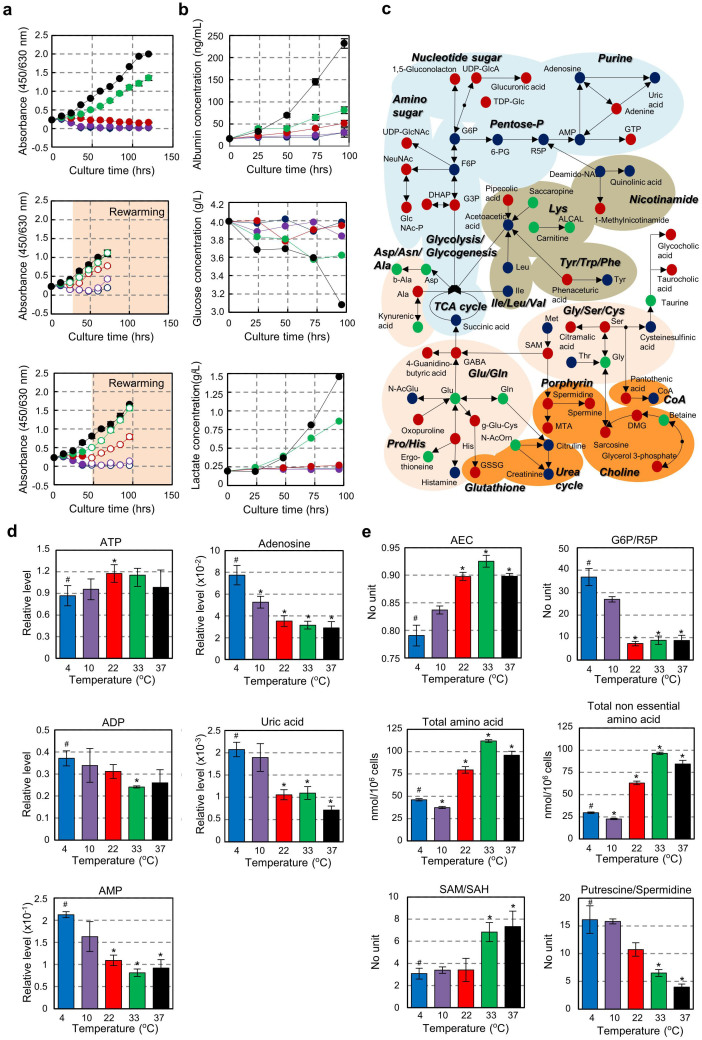
Affects on cellular activity and intracellular metabolism due to temperature change. (a), Assessments of proliferative activity in Huh7 cells at various culture temperatures (*top*) and re-warming to 37°C from each culture temperature at 24 hrs (*middle*) or 48 hrs (*bottom*). The coloured lines represent temperatures of 37°C (black), 33°C (green), 22°C (red), 10°C (purple) and 4°C (blue). (b), Measurements of albumin synthesis (*upper*), glucose concentration (*middle*) and lactate concentration (*bottom*) in the culture supernatant. The coloured lines represent temperatures of 37°C (black), 33°C (green), 22°C (red), 10°C (purple) and 4°C (blue). (c), Schematic representation of the intercellular metabolic pathways in primary hepatocytes at culture temperatures of 4, 10, 22, 33 and 37°C. The accumulation of each metabolite is indicated by coloured dots, which are highly expressed in the lower temperature condition (4, 10°C; blue), the hypothermic and body temperature condition (22, 33, 37°C; red). The dependence with temperature increases (green). (d), Relative levels of ATP (*top left*), ADP (*middle left*), AMP (*bottom left*), adenosine (*top right*) and uric acid (*middle right*) in primary hepatocytes under various culture temperatures. The coloured bars represent temperatures of 37°C (black), 33°C (green), 22°C (red), 10°C (purple) and 4°C (blue). *p < 0.05, *vs.* mean value of 4°C condition (#) by *t*-test. (e), Relative levels of adenylate energy charge (AEC; *top left*), glucose 6-phosphate/ribose 5-phosphate (G6P/R5P; *top right*), total amino acids (*middle left*), total non-essential amino acids (*middle right*), *S*-adenosylmethionine/*S-* adenosyl-L-homocysteine (SAM/SAH; *bottom left*) and putrescine/spermidine (*bottom right*). The coloured represent temperatures of 37°C (black), 33°C (green), 22°C (red), 10°C (purple) and 4°C (blue). *p < 0.05, *vs.* mean value of 4°C condition (#) by *t*-test.

To investigate the effect of the culture temperature on cellular activities including cell proliferation, albumin synthesis and glucose metabolism of the liver, we analysed the responses of a human hepatocellular cell line, Huh7, under various culture temperature conditions, and we used these cells as a model of the hepatocytes that constitute the majority of the liver. Cell proliferation at 37 and 33°C was clearly temperature-dependent, whereas proliferation below 22°C could not be observed (Fig. 2a, *top* and Supplementary Fig. 2a). The cells cultured at 22°C, but not those at 4 and 10°C, could successfully maintain the ability to proliferate in response to rewarming to 37°C for at least 48 hrs (Fig. 2a, *centre* and *bottom*). Albumin synthesis and glucose metabolism, which produces lactate from glucose and is related to cell proliferation and survival, were also observed in a temperature-dependent manner at 33 and 37°C, according to the results of the cell proliferation assay (Fig. 2b). These results indicate that the culture temperature affects cellular activities, and the marginal temperature for cell survival might occur at approximately 22°C.

### Functional analysis of intracellular metabolism changes due to temperature change

To analyse the intracellular metabolism as a function of culture temperature, we performed a metabolome analysis of primary cultured hepatocytes under various culture temperatures. In this study, the major metabolites relevant to hepatocellular metabolism were analysed (Supplementary Table 1). In a heat-map analysis of intracellular metabolites under various culture conditions, the metabolome profiles of the 4 and 10°C culture conditions were clearly different to those of the other temperature conditions (Supplementary Fig. 2b). The relative concentrations of metabolites in all culture conditions were analysed statistically and then divided into four groups. Group 1 included the higher metabolite accumulation at the 22, 33 and 37°C culture conditions compared with the 4 and 10°C cultures. Group 2 included the higher metabolite accumulation at the 4 and 10°C culture conditions compared with the 22, 33 and 37°C cultures. Group 3 included the accumulated metabolites dependent on the temperature rise. And Group 4 included the metabolites with relative concentrations that were not detectable at each culture temperature (Supplementary Table 1).

The categorised metabolites in Groups 1 to 3 are summarised in Table 1 and shown in the representative metabolic pathway maps in Fig. 2c and Supplementary Fig. 3. The accumulation of decomposition products of a nucleotide phosphate, such as adenosine monophosphate (AMP), adenosine and uric acid, which are the end products of purine metabolism, was detected in the hepatocytes cultured at 4 and 10°C (Fig. 2d). These results suggest that the intracellular energy metabolism was significantly preceded by ATP decomposition compared with the 22, 33 and 37°C conditions. The relative ratio of adenylate energy charge (AEC), which is an energy synthetic marker^22^, was significantly reduced compared with the 4 and 10°C temperature conditions (Fig. 2e). From an analysis of glucose-6-phosphate/ribose 5-phosphate (G6P/R5P), which is a preferential progression of the glycolytic pathway, the amount of G6P for the 4 and 10°C culture conditions were found to be at significantly high levels compared with those in the cells at 22–37°C culture conditions. These results indicate that the cells could not metabolise the glycolytic substrates normally (Fig. 2e). The amount of total and non-essential amino acids also increased in the culture conditions at 22, 33 and 37°C (Fig. 2e). The hepatocytes cultured at 37 and 33°C clearly exhibited high levels of S-adenosylmethionine/S-adenosyl-L-homocysteine (SAM/SAH), which is a DNA methylation molecule that is used for gene expression and for the functional regulation of proteins through methylation^23^. The SAM levels were significantly higher at 37 and 33°C than at 4–22°C. In addition, the amount of spermidine, which is involved in cellular proliferation and produced by putrescine and SAM^24^, was also significantly increased at 22, 33 and 37°C (Fig. 2e). Furthermore, hepatocyte-specific functions, including the urea cycle, bile production and phospholipid synthesis, which are also associated with cell proliferation, were affected in temperature-dependent manner but were suppressed at 4 and 10°C (Fig. 2c and Table 1). These results indicate that intracellular metabolism was strongly regulated by temperature. Therefore, the hypothermic conditions at approximately 22°C served as a biological temperature for harmonious cellular activities, including energy synthesis and cell functionality.

### Functional replacement by the transplantation of a cultured liver

**Figure 3:**
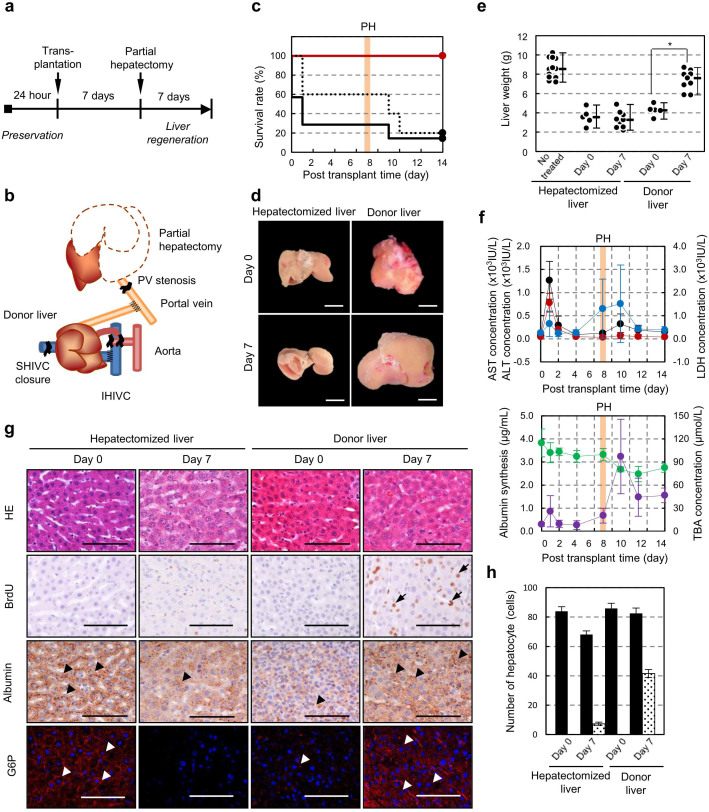
Functional replacement by cultured liver transplantation. (a), Time course representation of perfusion culture and transplantation protocol. (b), Schematic representation of auxiliary liver transplantation method modified from our previously reported method^42^, the 70% partial hepatectomy and the portal vein stenosis. (c), Survival rate after transplantation using the cold-preserved liver with UW solution (dot line, n = 5), the cultured liver with erythrocytes (red line, n = 9) and the cultured liver without erythrocytes (black line, n = 7). Seven days after transplantation, a partial hepatectomy (PH) and PV stenosis (orange line) were performed. Statistical analyses were performed at 14 days. *p < 0.05, *vs.* cold preserved group by *t*-test. (d), Photographs of the hepatectomised host liver and transplanted donor liver at 0 and 7 days after PH and PV stenosis. Scale bars: 1 cm. (e), Liver weight after partial hepatectomy and PV stenosis. The dots represent the weights of the untreated recipient's liver (n = 12), host liver at 0 days (n = 5) and 7 days (n = 11), transplanted liver at 0 days (n = 6) and 7 days (n = 12). The data are shown as the median ± max. Statistical analysis was performed by a log-rank test using the statistical software Prism 6. (f), Assessments of serum markers after transplantation. These data are represented by ALT (red), AST (black), LDH (blue) in the top graph and albumin (green) and TBA (purple) in the bottom graph. (g), Histological analysis (*top*) and immunohistochemical analysis including Brd-U (*second columns*), albumin (*third columns*) and G6P(red; *lower*) of the hepatectomised liver (*left and centre left*) and transplanted liver (*centre right and right*) at 0 and 7 days after PH and PV stenosis. The G6P sections were counterstained with Hoechst 33342 (blue). BrdU positive cells (arrow), albumin positive cells (black arrowhead) and G6P positive cells (white arrowhead) were observed. Scale bars: 100 μm. (h), Assessment of the number of Brd-U positive hepatocytes (dotted bar) and total hepatocytes (dark bar) of the host and transplanted livers at 0 and 7 days after PH and PV stenosis. These data are shown as the mean ± s.e.m. (*n* = 20).

We next investigated whether the cultured liver in our perfusion culture system showed potential for functional replacement with a recipient's liver and regeneration after partial hepatectomy (PH) (Fig. 3a). We performed donor liver preservation for 24 hrs using three experimental groups: static cold storage at 4°C and our perfusion culture with or without erythrocytes at 22°C. All of the donor livers were transplanted into unilateral nephrectomised rats via an auxiliary liver transplantation with arterial reconstruction. PH of the recipient's liver, which involved resection of the left and median lobes and constriction of the hepatic portal vein, was performed 7 days after transplantation to analyse the liver regeneration potential of the cultured liver (Fig. 3b). The survival rates of the rats that received static cold storage livers or livers in culture without erythrocytes immediately decreased after transplantation, and the survival rate after PH eventually decreased to less than 20% (Fig. 3c). By contrast, the survival rate of rats that received livers cultured using our perfusion system with erythrocytes significantly increased (*p* = 0.0005) (Fig. 3c). We analysed the liver weight and serum markers of rats that received transplanted cultured liver with erythrocytes after 24 hrs under 22°C perfusion. The liver weights at 7 days after PH also significantly increased from 4.26 ± 0.60 g 7 days after transplantation to 7.38 ± 1.04 g, which was equivalent to that of the untreated host liver (8.22 ± 1.06 g) (Figs. 3d, e). The concentration of lactate dehydrogenase (LDH) as a general disorder marker in the recipient serum transiently increased during the surgical procedures, including liver transplantation and PH. By contrast, alanine aminotransferase (ALT) and aspartate aminotransferase (AST) as hepatic disorder markers exhibited a transient increase immediately after transplantation; these markers then decreased at subsequent observation periods (Fig. 3f, *top*). The concentration of albumin, which decreased after PH, increased within 7 days after PH (Fig. 3f, *bottom*). The concentration of total bile acid (TBA), which is related to lipid synthesis, transiently increased after PH and then decreased during the transplantation period (Fig. 3f, *bottom*). The decrease of the TBA concentration was due to the recovery of bile acid secretion from the transplanted liver to the gastrointestinal tract. The cultured livers after transplantation showed almost no histological damage related to ischaemia-reperfusion injury (IRI) in the portal vein epithelium, arterial endothelium and bile duct. The hepatocyte cell growth of the cultured liver with erythrocytes at 22°C was observed by bromodeoxyuridine (BrdU)-labelling (Fig. 3g, h). The number of BrdU-positive hepatocytes was significantly increased in the cultured liver after PH, whereas in the host liver, in which the portal vein was partially ligated to prevent liver regeneration, the number of BrdU-positive hepatocytes was slightly increased (Fig. 3h). Immunohistochemical analyses revealed that albumin and glucose 6-phosphatase (G6P)-positive hepatocytes drastically increased after PH compared with the host liver (Fig. 3g). These results indicate that our perfusion culture system with erythrocytes of 22°C culture successfully maintained the function and regeneration potential of the liver.

### Resuscitation of DCD liver using our perfusion culture system

**Figure 4:**
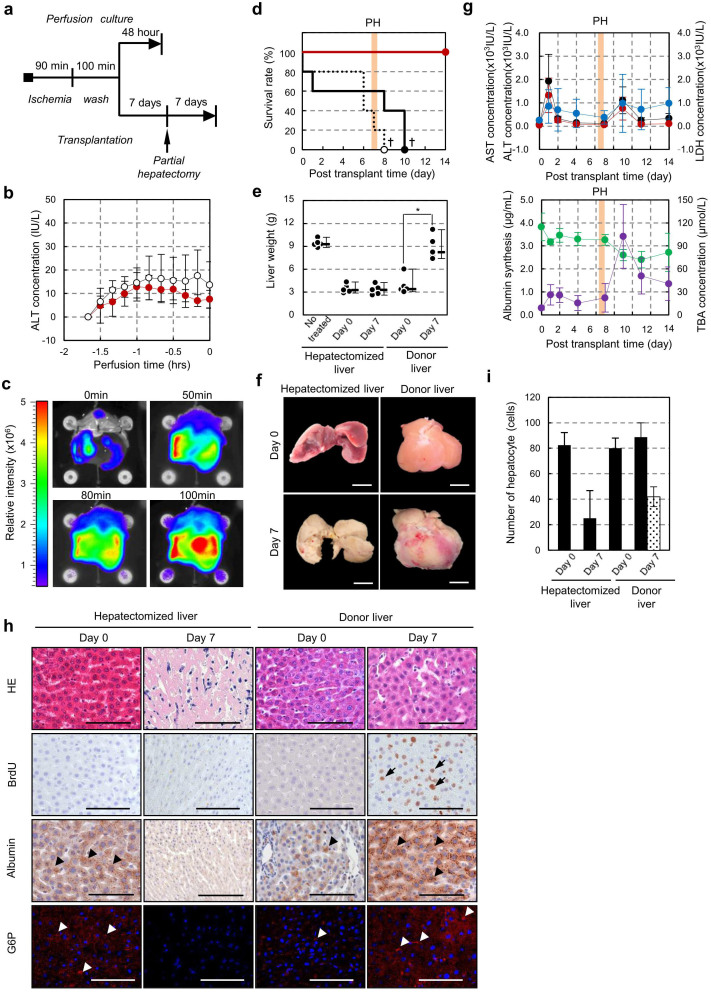
Resuscitation of DCD liver by normothermic perfusion culture. (a), Time course representation of perfusion culture and surgical procedure in DCD liver. (b), Assessment of ALT activity in DCD liver during normothermic perfusion with 5.0 × 10^11^ cells/L erythrocytes (red) and without erythrocytes (white). (c), Photographs of ATP accumulation in DCD liver using the perfusion culture with 5.0 × 10^11^ cells/L erythrocytes under 22°C. (d), Survival rate after transplantation using cold preserved DCD liver with UW solution (dot line, n = 5), cultured DCD liver with erythrocytes (red line, n = 5) and a cultured DCD liver without erythrocytes (black line, n = 5). Seven days after transplantation, PH and PV ligation (orange line) were performed. Statistical analysis was performed by a log-rank test using the statistical software Prism 6. (e), Liver weight after PH and PV ligation. Dots represent the weights of the untreated recipient's liver (n = 5), hepatectomized host liver at 0 day (n = 5) and 7 days (n = 5), transplanted liver at 0 day (n = 5) and 7 days (n = 6). The data are shown as the median ± max. *p < 0.05 by *t*-test. (f), Photographs of the hepatectomised liver and transplanted donor liver at 0 and 7 days after PH and PV ligation. Scale bars: 1 cm. (g), The serum markers after transplantation. These data are represented by ALT (red), AST (black), LDH (blue) in the top graph and albumin (green) and TBA (purple) in the bottom graph. (h), Histological analysis (*top*) and immunohistochemical analysis including Brd-U (*second columns*), albumin (*third columns*) and G6P (red; *lower*) of the hepatectomised liver (*left and centre left*) and transplanted liver (*centre right and right*) at 0 and 7 days after PH and PV ligation. The G6P sections were counterstained with Hoechst 33342 (blue). BrdU-positive cells (arrow), albumin-positive cells (black arrowhead) and G6P-positive cells (white arrowhead) were observed. Scale bars: 100 μm. (i), The number of Brd-U positive hepatocytes (dotted bar) and total hepatocytes (dark bar) of the livers at 0 and 7 days after PH and PV ligation. These data are shown as the mean ± s.e.m. (*n* = 20).

DCD livers are known to be damaged by ischaemia and have not been sufficiently utilised in liver transplantation^16^. We investigated whether our optimised perfusion culture system could be used for resuscitation of a DCD liver in a rat model. We performed our optimised perfusion culture without or with erythrocytes for 100 min using DCD livers, which were allowed to undergo cardiac arrest for 90 min at room temperature *in vivo*. All of the donor livers were transplanted via an auxiliary liver transplantation, and then PH of the recipient liver was performed at 7 days after transplantation (Fig. 4a). A significant difference was not observed in the ALT release in the initial perfusion culture period of 100 min when perfusion with and without erythrocytes were compared (Fig. 4b). By contrast, liver damage, as analysed by ALT concentration, salient swelling and disorganisation of the sinusoidal structure in the perfusion culture for 48 hrs, was maintained at a low level compared with the no-erythrocyte group (Supplementary Fig. 4a, c). Albumin was also produced continuously (Supplementary Fig. 4b). Histological changes in the perfused DCD liver without erythrocytes were observed with the vacuolation and nuclear fragment of hepatocytes, whereas the perfused livers with erythrocytes maintained a well preserved structure (Supplementary Fig. 4c). Based on the culture period, ATP in the DCD liver, which decreased after the 90-min cardiac arrest, accumulated after our perfusion culture with erythrocytes (Fig. 4c).

The survival rates of rats that received livers in static cold storage or livers cultured in our perfusion system without erythrocytes decreased even after the auxiliary transplantation, and all of the rats died after PH within 3 days (Fig. 4d). By contrast, the rats engrafted with the cultured liver using our perfusion culture with erythrocytes at 22°C showed a significant increase in the survival of recipients (*p* = 0.0018) (Fig. 4d). We analysed the liver weight and serum markers of the rats that received cultured DCD livers that were perfused with erythrocytes for 100 min at 22°C. The weights of these grafts at 7 days after PH significantly increased from 3.90 ± 1.20 g of the liver after the transplantation for 7 days to 8.85 ± 1.56 g, which is equivalent to the weights of the untreated wild-type livers of 9.40 ± 0.50 g; the recipient's liver that was ligated completely to prevent liver regeneration could not increase (Figs. 4e, f). The concentrations of ALT, AST and LDH in the recipient serum transiently increased due to the surgical procedures, including liver transplantation and PH (Fig. 4g, *top*). The albumin concentration of the cultured liver decreased after PH and then slightly increased after 7 days. The TBA concentration of the rats engrafted with the cultured liver transiently increased after PH and subsequently decreased (Fig. 4g, *bottom*). The sinusoidal structure and hepatocytes were maintained in the cultured DCD liver with erythrocytes at 22°C, and BrdU-positive hepatocytes were clearly detected (Figs. 4h, i and Supplementary Fig. 3d). The host liver at 7 days after PH progressed to fibrosis of the liver tissue due to necrosis and a specific dysfunction, including albumin synthesis and gluconeogenesis (Figs. 4 h, i). Liver functions such as albumin and G6P expression were detected after transplantation of the cultured DCD liver, and these expressions dramatically increased 7 days after PH (Figs. 4 h, i). These results indicate that our optimised perfusion culture system can resuscitate a DCD liver that can be used for functional replacement and regeneration *in vivo*.

## Discussion

Here, we demonstrate the successful development of a 3D organ perfusion culture system that can be used for reliable preservation of a donor organ and the resuscitation of a DCD organ. This method can be used to reduce the donor organ shortage. By controlling the metabolic conditions, the cell proliferation and physiological function are suppressed at low levels even though the cellular metabolic functions necessary for survival are maintained. This study illustrates the potential for an *ex vivo* organ perfusion culture system for organ preservation and organ resuscitation that is optimised by the essential culture conditions underlying oxygenation and thermoregulation.

Humans and other mammals use thermoregulation to maintain a stable internal body temperature. This includes activities such as muscle shivering and increasing the metabolic rate to produce heat^12^, and thermoregulation plays essential roles in regulating the biological activities of cellular and organ functions, physiological responses and homeostasis in multicellular organisms^12^. Hibernation in some mammals is a unique survival strategy in which the metabolic and physiological conditions change at low body temperature, thereby enabling survival^26^,^27^. Hypothermia in humans is also a life-threating condition that decreases the heart rate and respiration in ways similar to hibernation^11^. The critical core temperature that separates survival and death in hypothermia has been reported to be approximately 20°C, and patients can be revived from severe hypothermic conditions using appropriate clinical treatment^28^,^29^. A cerebral hypothermia treatment, which involves moderate hypothermia between 31 and 33°C, has been used as a clinical treatment for severe brain damage^10^. We have previously reported that a kidney preservation model using normothermic storage at 23°C successfully prevented reperfusion injury^30^. In this study, we evaluated a novel hypothermic 3D perfusion culture using liver as the model organ, which is a difficult organ preservation model compared with the kidney model, and we successfully demonstrated that the hypothermic 3D perfusion culture at 22°C could suppress organ damage and maintain organ function. In this hypothermic cell culture, the primary hepatocytes could survive in the long term without cell proliferation, and they have the potential to revive cell proliferation and functions in response to an increase in temperature. These findings suggest that *ex vivo* regulation of temperature contributes to the establishment of an extracorporeal organ culture/organ development system.

Cellular metabolism, which is involved in both the catabolism of macromolecules and the synthesis of organic substances such as proteins, nucleic acids, polysaccharides and lipids, plays essential roles in various biological reactions. Endothermy and homeothermy rely on cellular metabolism to control the body temperature through the production of heat. It is known that temperature strongly regulates cellular metabolism. The metabolic flux of the glycolytic pathway leads to activation of the pentose phosphate pathway below 32°C^31^. The conventional static cold storage method that functions via the suppression of metabolic reactions in the low temperature range (4°C) is effective for short-term organ preservation^32^. In donor organ transplantation, the consumption of intracellular essential molecules such as glycolytic substrates, amino acids and nucleotide compounds at low temperature plays important roles in cell survival^33^. However, the failure of organ transplantation is caused by the depletion on energy substrates during long-term storage^33^. In this study, we demonstrated that hepatocellular metabolic reactions associated with cell survival and physiological activity at 22, 33 and 37°C were significantly maintained as indicated by the metabolome analysis results. In particular, the energy synthetic pathway at 22, 33 and 37°C (but not under low-temperature culture conditions) maintained a preferable balance of ATP synthesis/consumption. The levels of polyamines and SAM, which are needed for cell proliferation and methylation reactions, also exhibited a different tendency compared with the markers involved in energy production, such as AEC, amino acids and G6P/R5P, between 37°C and 22°C. Current approaches, including extracorporeal machine perfusion at normothermic conditions^34^,^35^ and brain hypothermic therapy at 30–33°C^10^, might be effective not only for maintaining the energy balance to prevent organ dysfunction but also for the suppression of various cellular functions associated with the consumption of energy substrates. These results suggest that organs stored long term at low temperature cannot be revived because of irreversible changes in the energy synthesis components that are needed for cell survival. Thus, this study suggests that the cellular responses of cultivation temperature based on metabolic mechanisms should be elucidated for the development of an extracorporeal organ culture system that mimics an *in vivo* environment.

Donor organ transplantation is traditionally performed to replace a dysfunctional organ^13^,^18^; however, the current static cold storage systems, which aim to prevent damage to the donor organ, are not sufficient for long-term preservation or pre-treatment of organ conditions^36^,^37^. 3D organs must receive sufficient oxygen and nutrients from the 3D vascular network for the maintenance of physiological organ functions such as exocrine and endocrine functions, digestion and absorption, metabolism and waste release^38^,^39^. These activities are slowed down as the temperature decreases, and only the minimum functions for survival are able to continue, as occurs during hibernation^40^. Current organ preservation methods strongly inhibit organ metabolism to prevent consumption of the essential substrates and supress the minimum functions for cellular survival, such as ATP synthesis and amino acid metabolism^12^,^33^. In this study, we developed a useful extracorporeal organ perfusion culture system that could be used for highly efficient oxygenation with erythrocytes and can be used for optimisation of the culture temperature to support biological metabolism. This system also showed potential for preventing organ damage and dysfunction in organ transplantation. We also demonstrated that our perfusion culture could contribute to the resuscitation of DCD organs that have severe ischaemic injuries. Furthermore, our organ perfusion culture system performed at approximately 22°C and allowed *ex vivo* recovery of ATP production by maintaining organ metabolism. To maintain the organ outside the body, it would be effective to induce the organ into a “static low activated state” to maintain the minimum metabolic activity by regulating the temperature at 22–23°C and also supplying oxygen by proper aerobic respiration. These findings represent a significant advancement in organ preservation/resuscitation technology for organ transplantation, and they show further potential for *ex vivo* 3D organ cultivation and organ development in the future.

In conclusion, our study demonstrated the establishment of a novel organ perfusion culture system in which donor organ preservation was achieved by optimising the oxygen supply and culture temperature for biological cellular activity. Our extracorporeal culture system also represents a significant advancement for therapeutic concepts that involve resuscitation of severely ischaemic organs, thus contributing to the reduction of the donor organ shortage. Additional studies on ways to improveme the culture solution and system regulation, including validation of the temperature for maximum cell activation, will contribute to the future development of 3D organ development systems.

## Methods

### Animals

Male Wistar rats weighing 150–200 g were purchased from SLC Inc. (Shizuoka, Japan), and LEW-Tg (Gt(ROSA)26Sor-luc)11Jmsk were kindly provided by Dr E. Kobayashi^41^. All handling and care of the rats conformed to the National Institutes of Health (NIH) guidelines for animal research, and all experimental protocols involving animals were approved by the Tokyo University of Science Animal Care and Use Committee (Permit Number: N13040). All experiments involving animals were carried out in accordance with these guidelines and experimental protocols. All efforts were made to minimise suffering.

### Liver isolation procedure

Donor rats were anaesthetised using an inhalation anaesthesia apparatus (Univentor, Zejtun, Malta) with isoflurane concentrations of 1.5% to 3% and an air flow of 0.4 L/min. After a tail vein injection of 2500 U heparin sodium (Wako, Osaka, Japan), a 22 G catheter (Surflo F&F, Terumo, Tokyo, Japan) was inserted into the portal vein (PV) and bile duct, and a 18 G catheter (Terumo) was inserted into the suprahepatic inferior vena cava (SHIVC). In the rat livers, perfusion of the culture medium from the PV cannula was initiated, and the livers were harvested with the surrounding tissues, including the diaphragm and costal arch.

### Extracorporeal organ perfusion culture system

Our extracorporeal organ perfusion culture system consisted of a perfusate reservoir (ABLE, Tokyo, Japan), an organ chamber (ABLE) and bio pumps (IWAKI, Tokyo, Japan) on a clean bench. This system could maintain a constant culture temperature (minimum, 0°C; maximum, 50°C). The perfusate reservoir contained an oxygen sensor, a pH sensor and an oxygenator for regulation of the oxygen supply (dissolved oxygen concentration (DO); 6.77). An isolated liver was placed in the organ chamber by hanging with the costal arch and floating in leivobits-15 culture medium (L-15; Sigma-Aldrich Japan, Tokyo, Japan; KOJIN BIO, Saitama, Japan). This system required 1,000 mL of the perfusion culture solution to run the circuit. The culture solution consisted of L-15 culture medium containing 10% FBS, 1% antibiotic-antimycotic mixed stock solution (Nacalai tesque, Kyoto, Japan), 25 mg/L gentamycin (Wako), 0.292 g/L L-glutamine (GIBCO, Grand Island, NY, USA), 3 mg/L cyclosporine A (Wako), 50,000 U/L heparin (Wako), and human erythrocytes as oxygen carriers (transferred from the Japanese Red Cross Society, Tokyo, Japan). The isolated liver was perfused with oxygenated culture solution at a flow rate of 11 mL/min through the PV, and perfusion outflow was collected from the SHIVC. The culture solution was continuously warmed in a heat exchanger to maintain the temperature at 37°C.

### Biochemical analysis of liver perfusion culture

Culture solution samples were collected in the culture circuit. Alanine aminotransferase (ALT) was analysed as an indicator of liver damage, and the bile secretion, albumin synthesis and urea production were analysed as indicators of liver physiological functions. The ALT concentration was measured by using the transaminase CII Kit (Wako). The albumin concentration was determined by the enzyme-linked immunosorbent assay (Rat Albumin ELISA Quantitation Set; Bethyl Laboratories, Montgomery, USA). Bile secretion was also measured by automatic collection through the catheter connected to the bile duct every 4 hrs. To analyse urea production, cleansing perfusion of the cultured liver was performed for 2 hrs using a Krebs-Henseleit buffer, and the liver was loaded with 1 mM ammonia for 30 min. Effluent buffer samples were collected every 3 min, and the urea concentrations in the supernatants were measured using the ammonia urea F-kit (JK International, Tokyo, Japan).

### Histochemical analysis and immunohistochemistry

The natural liver and cultured liver at the end of perfusion were flushed with saline and fixed with 10% formalin (Mildfolm 10N; Wako). Hepatic tissues were obtained from the intermediate lobe. Tissue sections (5 μm thick) were taken after paraffin embedding and staining with haematoxylin and eosin (HE). Stained sections were observed using an Axioimager A1 microscope (Carl Zeiss, Oberkochen, Germany) and an AxioCAM MRc5 (Carl Zeiss). For immunohistochemistry, 10 μm frozen sections for G6P staining and 10 μm paraffin sections for albumin and Brd-U staining were prepared and immunostained as previously described. The specific antibodies used were anti-glucose-6-phosphatase, catalytic subunit antibodies (αG6P pAb; 1:200, Abcam, Cambridge, UK). The G6P immunoreactivity was detected using goat anti-rabbit IgG(H+L) Alexa Fluor 594 highly cross-absorbed molecular probe (pAb; 1:500, Chemicon) counter-stained with Hoechst 33342 (pAb; 1:500, Hoechst AG, FRG). The albumin specific antibodies used were HRP-conjugated rat albumin detection antibodies (Bethyl Laboratories, Alabama, US). The specific antibody used was a POD-conjugated mouse Brd-U detection antibody (Roche, Basel, Switzerland). All fluorescence images were obtained using a confocal laser microscope (LSM 780, Carl Zeiss).

### Analysis of cell proliferation activity of transplanted livers

The recipients were injected with 12.5 mg/mL Brd-U (i.p) for 7 days after PH, and the livers were removed for immunostaining. Hepatocytes and Brd-U positive hepatocytes were counted in each sample image.

### Analysis of the angiographic images using propidium iodide staining and FITC-gelatine conjugate

To visualise the sinusoidal structure after liver perfusion culture, we prepared a solution of fluorescein 5-isothiocyanate (FITC; Dojindo, Kumamoto, Japan) conjugated to gelatine (Sigma-Aldrich Japan) and dissolved in 1 mL dimethylsulfoxide (DMSO; Sigma-Aldrich Japan) at pH 11. The FITC solution and 20% (w/v) gelatin solution were mixed for conjugation at 37°C overnight. The unconjugated FITC was removed using a NAP-25 column (GE Healthcare UK Ltd., Buckinghamshire, England). To identify dead cells, propidium iodide (PI) staining was performed by perfusion of L-15 medium containing 5 μg/mL PI for 20 min. After flushing with saline, 25 mL of filtrated FITC-conjugated gelatin was perfused via the portal vein and fixed by Zamboni solution at freezing temperature. From these samples, frozen sections (10 or 100 μm thick) were prepared after the fixation using 8% formalin containing Hoechst 33342 dye (1:500, Life Technologies, Carlsbad, CA, USA). These fixed sections were washed with PBS (-) and examined using laser confocal microscopy (LSM780, Carl Zeiss). Areas of 800 μm (wide) × 200 μm (depth) × 100 μm (height) were observed. In these 3D images, the area between the PV and CV was divided into five equal parts as a range to 100 μm (wide) × 200 μm (depth) × 100 μm (height), and all hepatocytes and PI positive cells in each area were counted using IMARIS software.

### Liver ischaemic condition and resuscitative procedure by perfusion culture

Donor rats were euthanised by cervical dislocation and underwent 90-min cardiac arrest at room temperature. Then, an ischaemic liver was isolated and connected to our perfusion culture system in the same manner as a fresh liver. In the 100-min perfusion culture for the ischaemic liver, the ALT concentration of the effluent was evaluated as an indicator of organ damage. We also analysed the amount of internal ATP in the resurrected liver using the non-invasive bio-imaging system IVIS (Xenogen, Alameda, CA, USA) and the IVIS Living Image (Xenogen) software package. Luc-Tg rats were treated by ischaemic conditioning, and the livers of the perfusion circuit were established in the same manner. During the resuscitative procedure, we injected 3 mL of D-luciferin dissolved and diluted to 15 mg/mL in PBS (Promega, Madison, WI, USA) into the perfusate directly before the liver to detect the luminescence from the Luc-Tg liver. The signal intensity was quantified as photon flux in units of photons/s/cm^2^ of a steradian in the region of interest. After the resuscitative procedure by perfusion culture, these livers were transplanted to the recipient rats using the liver auxiliary transplantation method.

### Auxiliary liver transplantation

In this study, cultured livers were transplanted in an auxiliary manner with aortic reconstruction using a cuff technique modified using our previously reported method^42^. The right renal artery and vein of the recipient rat were isolated, and the right ureter was transected. After clamping the right renal artery and vein at their origin, the nephrectomy was performed. The cultured liver was placed in the right renal space of the recipient. A plastic cuff (22G Surflo F&F; Terumo) was inserted on the recipient's right renal artery. The donor hepatic artery and infrahepatic IVC were anastomosed to the recipient's right renal artery and vein, and the suprahepatic IVC of the donor liver was ligated. The recipient portal vein was connected to the donor portal vein with end-to-side anastomosis. The bile duct of the donor liver was connected to the recipient jejunum with a 2-mm-diameter stent (22G Surflo F&F; Terumo).

### Partial hepatectomy and portal vein ligation

To evaluate the functionality of the transplanted liver *in vivo*, a partial hepatectomy of the recipient liver was performed 7 days after auxiliary transplantation. Transverse abdominal incision was performed on the transplanted rats under anaesthesia, and the left lateral/intermedial lobes of the recipient's liver were resected. Continuously, the portal vein was ligated partially for cultured liver transplantation and ligated entirely for resuscitated ischaemic liver transplantation. After these procedures, the survival rate and the weight of the transplanted liver were analysed individually.

### Preparation of hepatocytes by collagenase perfusion method

The preparation of hepatocytes was performed by using the collagenase perfusion method as described previously. Abdominal incisions were performed on the rats under anaesthesia, and 18G catheters (Terumo) were inserted into the PV and IVC. The initial perfusions (100 mL; flow rate, 10–15 mL/min) were performed using the perfusion solution, which included NaCl (136.89 mM, Wako), KCl (5.37 mM, Wako), NaH_2_PO_4_/2H_2_O (0.38 mM, Wako), Na_2_HPO_4_/12H_2_O (0.17 mM, Wako), HEPES (10 mM, DOJINDO), glucose (5 mM, Wako), ethylene glycol tetra-acetic acid (0.5 mM, DOJINDO), NaHCO_3_ (4.16 mM, Wako), and phenol red (0.017 mM, Sigma-Aldrich Japan).

A secondary perfusion was performed for 8–15 min using collagenase solution (Wako), which contained NaCl (136.89 mM, Wako), KCl (5.36 mM, Wako), NaH_2_PO_4_/2H_2_O (0.38 mM, Wako), Na_2_HPO_4_/12H_2_O (0.17 mM, Wako), CaCl_2_ (5.06 nM, Wako), HEPES (10 mM, DOJINDO), glucose (5 mM, Wako), NaHCO_3_ (4.16 mM, Wako), phenol red (0.017 mM, Sigma-Aldrich Japan), and soy bean trypsin inhibitor (0.0025 mM, Sigma-Aldrich Japan). After the secondary perfusion, the liver was split into multiple tissues, and the primary hepatocytes were corrected through mesh filtration (Kawamoto, Osaka, Japan) in 2% FBS MEM.

### Cell culture

Huh7 cells were cultured in DMEM (KOJIN BIO) containing 10% FBS under highly humidified 95% air and 5% CO_2_. Cell morphological examination in each culture temperature was performed using a phase microscope (Carl Zeiss). A cell proliferation assay was performed using a WST-8 cell-counting kit (Dojindo,) and an albumin synthesis assay was performed by using an enzyme-linked immunosorbent assay (Rat Albumin ELISA Quantitation Set; Bethyl Laboratories,).

### Cell proliferative activity under culture temperature change

Huh7 cells were seeded into a 96-well plate (BD, Franklin Lakes, NJ, USA). After the initial culture at 37°C for 18 hrs, these cells were cultured under several temperatures (4, 10, 22, 33 and 37°C) for 24, 48 and 72 hrs. To analyse changes in cell proliferation activity due to temperature change, the cells at each temperature were re-warmed to 37°C. Viable cells in each culture were examined by using a WST-8 cell-counting kit (Dojindo). After each culture period, the cultured cells were incubated with WST-8 solution for 180 min, and the absorbance was measured at 450 nm with a reference wavelength at 630 nm.

### Metabolome analysis of culture hepatocytes by CE-TOFMS

Primary hepatocytes were seeded at a density of 1 × 10^5^ cells/cm^2^ and cultured initially at 37°C for 18 hrs with Williams' E medium (Sigma-Aldrich Japan) containing 10% FBS, 7 mM insulin (Wako), 7 mM dexamethasone (Sigma-Aldrich Japan) and 10 ng/mL human EGF (Peprotech). After the initial culture periods, these cells were cultured for 18 hrs at temperatures of 4, 10, 22, 33 and 37°C. To perform the metabolome analysis, hepatocytes were washed twice with 5% mannitol solution and were treated with 800 μL of methanol for 30 sec. The cell extracts were collected using 550 μL of Milli-Q water containing internal standards (H3304-1002, Human Metabolome Technologies, Inc., Tsuruoka, Japan) and centrifuged at 2,300 × *g* for 5 min. To remove some protein components, 800 μL of supernatants were filtered using a Millipore 5 kDa cut-off filter at 9,100 × *g* for 120 min at 4°C. Metabolome analysis using these samples was performed by a third-party service using CE-TOFMS (Human Metabolome Technology Inc.).

### Statistical classification of metabolites in hepatocytes

Our study classified the metabolites in hepatocytes to analyse intracellular metabolism comprehensively as a function of culture temperature. We used Welch's *t*-test for major metabolites, which were drawn in the metabolic pathway map, to determine *p*-values for the comparison of compound levels between low temperatures and high temperatures (mean values of the 4 and 10°C culture groups versus mean values of the 22, 33 and 37°C culture groups). We classified the significantly higher metabolite accumulation of the 22, 33 and 37°C culture conditions compared with 4 and 10°C culture as Group 1, and Group 2 consisted of higher metabolite accumulation in the 4 and 10°C culture conditions compared with the 22, 33 and 37°C cultures. Group 3 consisted of the accumulated metabolites dependent on the temperature increase, and Group 4 consisted of the metabolites whose relative concentrations were not significantly different at different temperature (Supplementary Table 1). The categorised metabolites that exhibited a 1.4-fold change or more in Groups 1 to 3 were summarised (Table 1) and arranged in a representative metabolic pathway map (Fig. 2c and Supplementary Fig. 3).

### Statistical analysis

Statistical significance was determined based on a two-tailed Welch's *t*-test. Survival rate analysis used Kaplan-Meier methods, and statistical analysis was performed by using log-rank tests in the statistical software Prism 6.

## Author Contributions

T.T. and E.K. designed the research plan; J.I., Y.M., F.I., R.S., J.P. and A.K. performed the experiments; J.I., M.O., N.K., T.M., Y.A., E.K. and T.T. developed novel assay systems and discussed the results; J.I., F.I., R.S., J.P., A.K. and M.K. analysed the data; and T.T., J.I. and M.O. wrote the paper.

## Supplementary Material

Supplementary Information Supplementary Information

## Figures and Tables

**Table 1:** Changes in specific metabolites in primary hepatocytes due to changes in culture temperature

Flux change between low and high temperature condition		
•(red) Accumulation at 22, 33, 37°C	•(blue) Accumulation at 4, 10°C	•(green) Increase with temperature rise
***Bile synthesis pathway***	***Glycolysis pathway***	***Amino acid metabolism pathway***
1-Methylnicotinamide	Fructose 6-phosphate	Asp
Glycocholic acid	Glucose 6-phosphate	Glu
Taurocholic acid		Gln
	***Penthose phosphate pathway***	Gly
***Glutathione metabolism pathway***	Ribose 5-phosphate	Saccharopine
5-Oxoproline	6-Phosphogluconic acid	Kynurenic acid
Glutathione		
	***Purine metabolism pathway***	***Cholin metabolism pathway***
***Purine metabolism pathway***	Adenosine	β-Ala
Adenine	AMP	Betaine
GTP		
	***TCA cycle pathway***	***Lipid, fatty acid metabolism pathway***
***Polyamine synthesis pathway***	Succinic acid	***O***-Acetylcarnitine
S-Adenosylmethionine		
GABA	***Urea cycle pathway***	***Urea cycle pathway***
Spermidine	Citrulline	N-Acetylornithine
Spermine	Creatinine	Ergothioneine
	N-Acetylglutamic acid	
***Urea cycle pathway***	Uric acid	***Bile synthesis pathway***
Sarcosine	Histamine	Carnitine
4-Guanidinobutyric acid		Taurine
5'-Deoxy-5'-methylthioadenosine	***Amino acid metabolism pathway***	
	Tyr	
***Sugar-nucleotide metabolism pathway***	Met	
UDP-glucuronic acid	Ile	
UDP-N-acetylglucosamine	Cysteinesulfinic acid	
Gluconolactone		
Glucuronic acid	***Ketone metabolism***	
dTDP-glucose	Acetoacetic acid	
***Amino-sugar metabolism pathway***	***Nicotinic acid metabolism pathway***	
*N*-Acetylglucosamine 1-phosphate	Quinolinic acid	
*N*-Acetylneuraminic acid		
***Glycolysis pathway***		
Glyceraldehyde 3-phosphate		
Dihydroxyacetone phosphate		
***Amino acid metabolism pathway***		
Ala		
Ser		
His		
Pipecolic acid		
*N*,*N*-Dimethylglycine		
***Lipid metabolism pathway***		
Glycerol 3-phosphate		
Pantothenic acid		

The metabolites that accumulated to high levels in primary hepatocytes are represented in the lower-temperature conditions (4, 10°C), the and the hypothermic and body temperature conditions (22, 33, 37°C), and the dependence on temperature increases. We represented the categorised metabolites by using a threshold value of 1.4-fold change or more in Groups 1 to 3.
