# Supplementary material for: Hypothermic temperature effects on organ survival and restoration
Source: Sci Rep. 2015 Apr 22;5:9563. doi: 10.1038/srep09563 (PMC4405701; doi:10.1038/srep09563)
Supplement: Supplementary Information [file srep09563-s1.pdf]

SUPPLEMENTARY INFORMATION

Hypothermic temperature effects on organ survival and restoration

Jun Ishikawa[1,2], Masamitsu Oshima[3], Fumitaka Iwasaki[1], Ryoji Suzuki[1], Joonhong Park[1], Kazuhisa Nakao[3], Yuki Matsuzawa-Adachi[1], Taro Mizutsuki[1], Ayaka Kobayashi[1], Yuta Abe[4], Eiji Kobayashi[5,6], Katsunari Tezuka[2,7] & Takashi Tsuji[2,3,7 *]

[1] *Department of Biological Science and Technology, Graduate School of Industrial Science and Technology, Tokyo University of Science, Noda, Chiba, 278-8510, JAPAN*

[2] *Laboratory for Organ Regeneration, RIKEN Center for Developmental Biology, Kobe, Hyogo, 650-0047, JAPAN*

[3] *Research Institute for Science and Technology, Tokyo University of Science, Noda, Chiba, 278-8510, JAPAN*

[4] *Department of Surgery, Keio University, School of Medicine, Shinanomachi, Shinjuku-ku, Tokyo, 160-8582, JAPAN*

[5] *Department of Organ Fabrication, Keio University, School of Medicine, Shinanomachi, Shinjuku-ku, Tokyo 160-8582, JAPAN*

[6] *Center for Development of Advanced Medical Technology, Jichi Medical University, Shimotsuke, Tochigi, 329-0431, JAPAN*

[7] *Organ Technologies Inc., Tokyo, 101-0048, JAPAN*

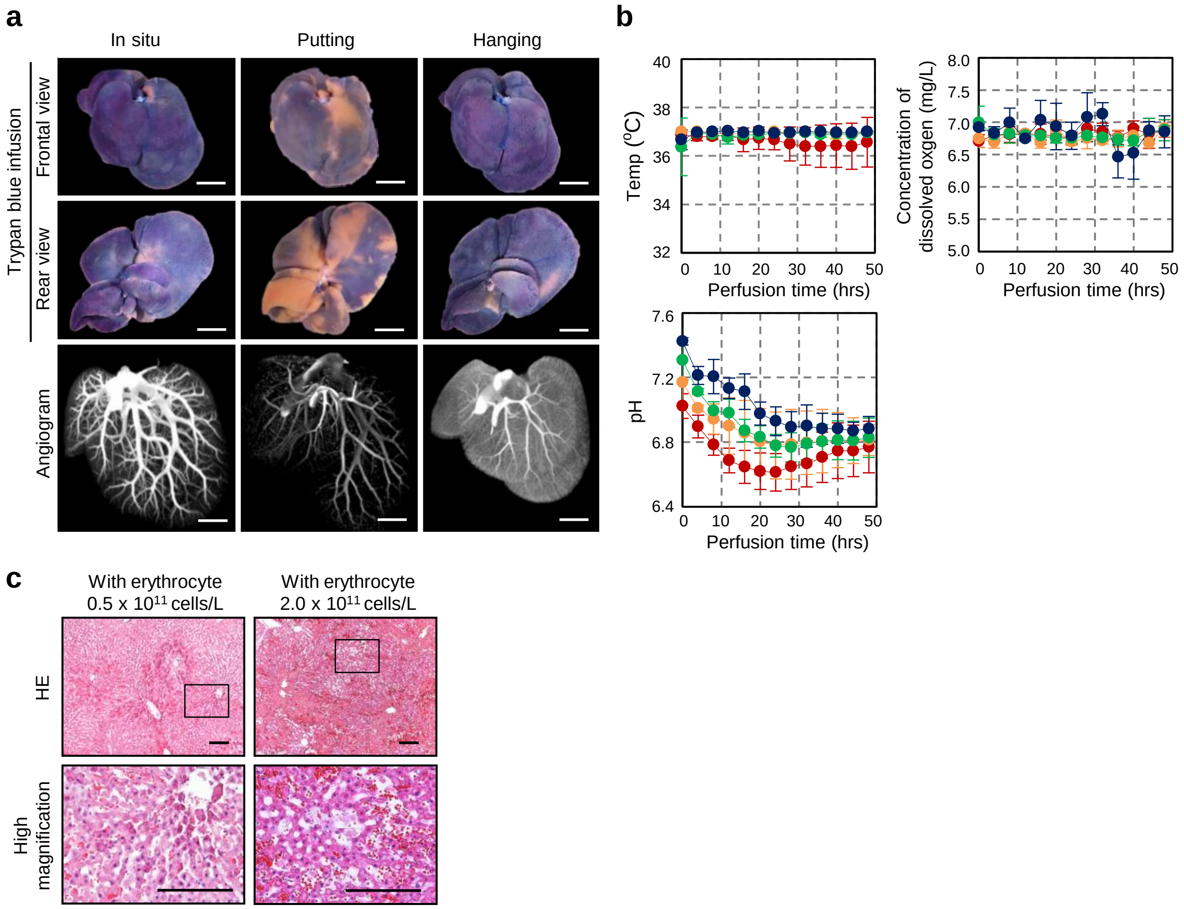

Supplementary Figure 1 J. Ishikawa *et al*.

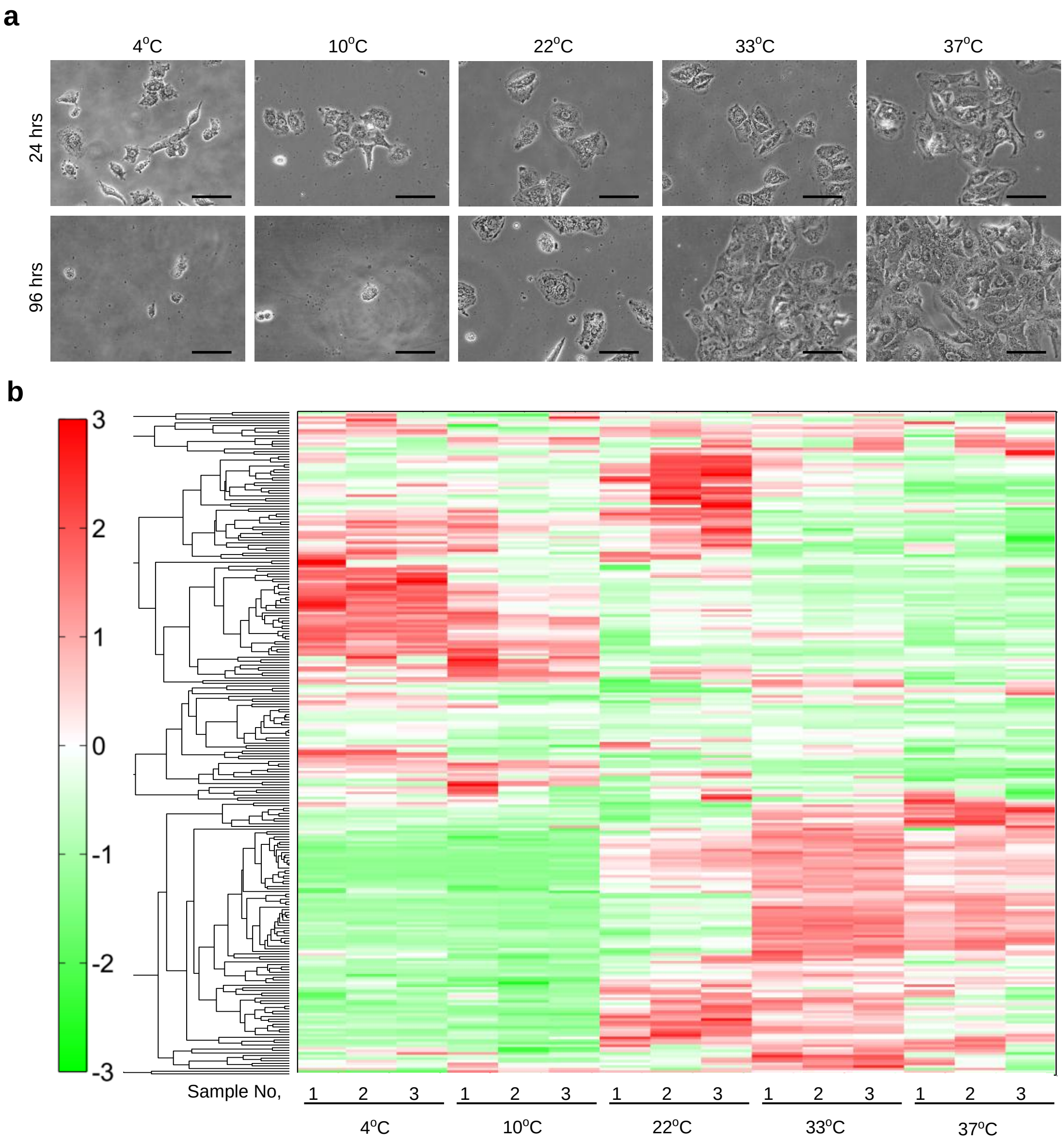

Supplementary Figure 2 J. Ishikawa *et al*.

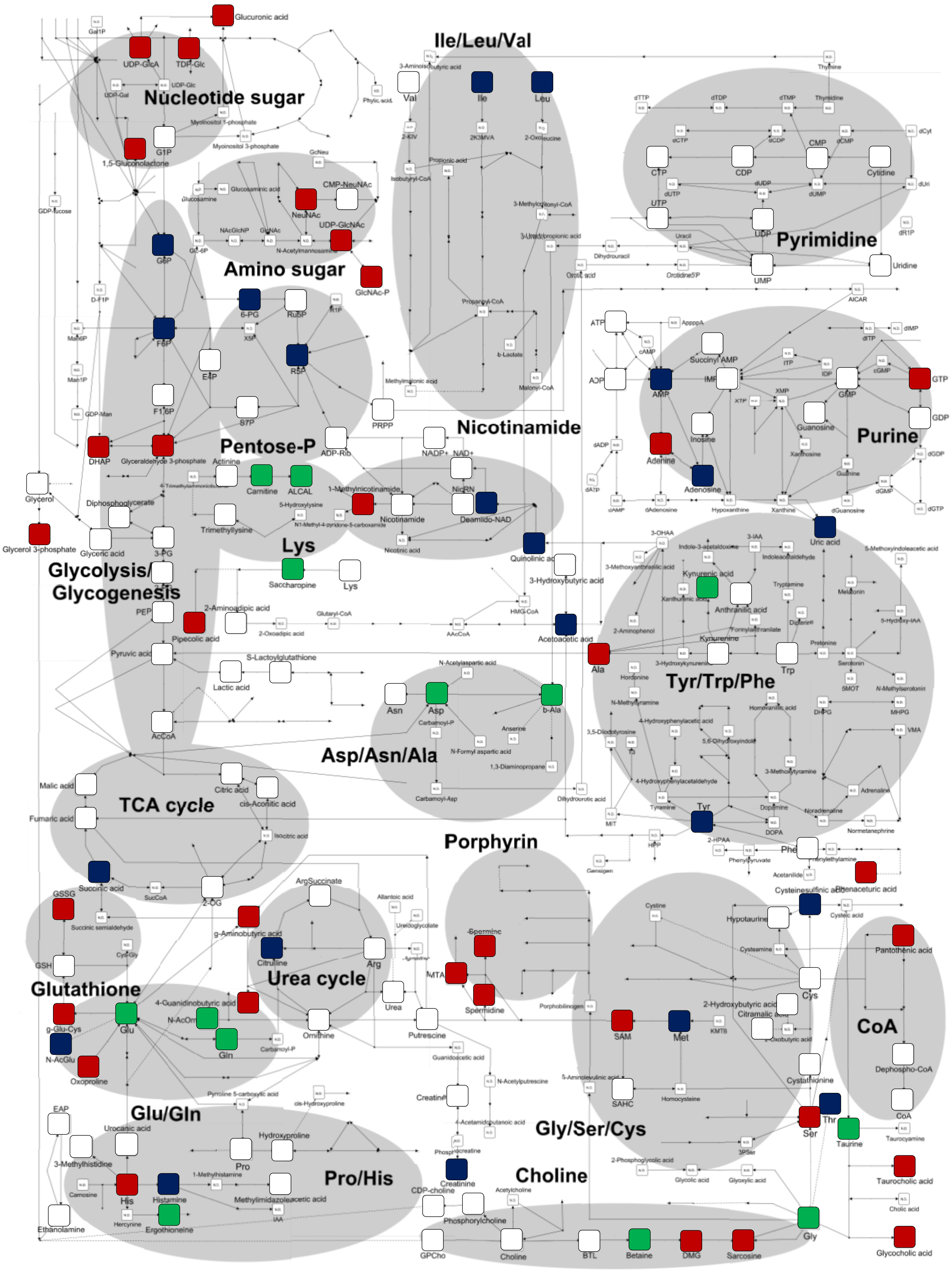

Supplementary Figure 3 J. Ishikawa *et al.*

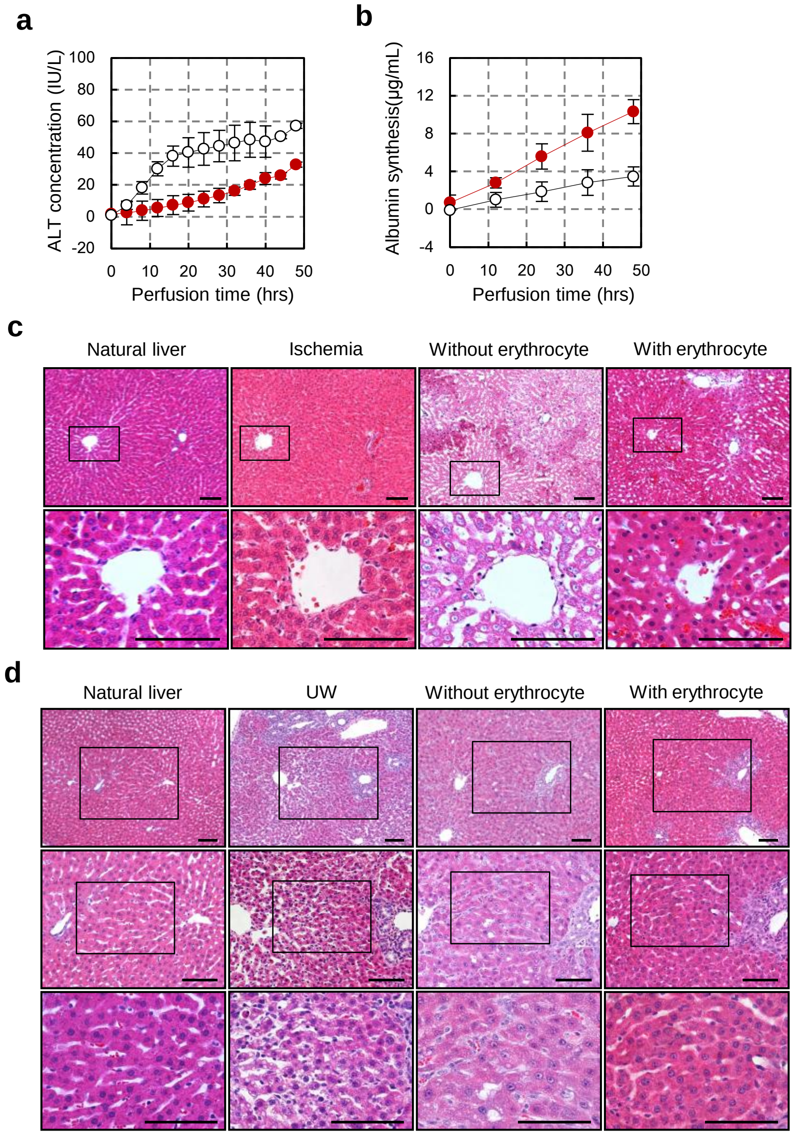

Supplementary Figure 4 J. Ishikawa *et al*.

	Compound name	Relative Area									
		4°C		10°C		22°C		33°C		37°C	
		Mean	S.D.	Mean	S.D.	Mean	S.D.	Mean	S.D.	Mean	S.D.
Group 1											
	Phenaceturic acid	2.1E-03	2.2E-04	2.2E-03	2.0E-04	3.0E-03	1.0E-04	3.1E-03	8.6E-05	2.1E-03	1.4E-04
	Pipecolic acid	1.9E-03	2.4E-04	1.9E-03	5.5E-05	5.5E-03	9.1E-04	3.3E-03	2.8E-04	2.6E-03	2.3E-04
	Dihydroxyacetone phosphate	2.1E-02	2.6E-03	1.3E-02	3.3E-03	3.9E-02	1.7E-03	3.3E-02	3.4E-03	2.6E-02	4.4E-03
	Ala	1.2E+00	5.6E-02	6.2E-01	1.7E-02	2.2E+00	1.8E-01	2.2E+00	1.2E-01	1.6E+00	6.6E-02
	1-Methylnicotinamide	3.6E-02	7.0E-04	3.7E-02	8.6E-04	6.0E-02	6.8E-03	5.9E-02	2.1E-03	4.5E-02	2.4E-03
	5'-Deoxy-5'-methylthioadenosine	3.1E-04	7.5E-05	3.5E-04	N.A.	5.3E-04	2.9E-05	8.4E-04	5.2E-05	5.9E-04	2.5E-05
	Ser	1.0E-01	5.7E-03	9.2E-02	5.8E-03	1.2E-01	1.4E-02	1.7E-01	1.5E-03	1.5E-01	1.1E-02
	GABA	2.4E-03	8.1E-05	2.1E-03	1.3E-04	1.0E-02	2.3E-03	8.5E-03	2.3E-04	6.5E-03	1.9E-04
	dTDP-glucose	6.0E-04	3.3E-05	5.6E-04	6.6E-06	8.8E-04	1.0E-04	8.7E-04	7.4E-05	7.3E-04	8.6E-05
	Glutathione (GSSG)_divalent	1.4E+00	9.5E-02	1.4E+00	1.8E-01	2.1E+00	3.7E-01	2.3E+00	2.5E-01	1.7E+00	6.6E-02
	GTP	4.8E-02	9.0E-03	5.8E-02	9.5E-03	9.6E-02	3.1E-03	8.2E-02	5.8E-03	7.5E-02	1.9E-02
	N-Acetylglucosamine 1-phosphate	2.3E-03	4.7E-04	3.1E-03	5.7E-04	5.2E-03	1.7E-03	5.8E-03	4.1E-04	3.6E-03	1.1E-03
	His	1.0E-01	8.8E-03	1.0E-01	2.2E-03	3.8E-01	4.6E-02	4.6E-01	6.4E-03	3.4E-01	1.9E-02
	5-Oxoproline	1.0E-02	6.2E-04	1.0E-02	2.0E-03	1.8E-02	3.5E-03	1.2E-02	1.5E-04	1.5E-02	3.4E-03
	Glycocholic acid	8.9E-03	6.3E-04	9.4E-03	7.0E-04	1.6E-02	3.6E-03	1.7E-02	1.6E-03	1.3E-02	2.0E-03
	Spermidine	1.8E-03	2.8E-04	1.8E-03	1.7E-04	4.1E-03	3.5E-04	4.8E-03	3.0E-04	3.8E-03	2.4E-04
	S-Adenosylmethionine	5.6E-03	4.5E-04	5.7E-03	4.6E-04	9.0E-03	6.7E-04	1.2E-02	3.1E-04	9.5E-03	5.3E-04
	N,*N*-Dimethylglycine	6.3E-03	3.8E-04	6.1E-03	4.1E-04	1.4E-02	2.4E-03	1.7E-02	1.7E-03	1.2E-02	3.3E-04
	Glyceraldehyde 3-phosphate	1.6E-03	5.5E-04	1.1E-03	3.1E-04	2.2E-03	3.6E-04	2.5E-03	2.0E-04	2.4E-03	2.5E-04
	UDP-glucuronic acid	6.3E-02	1.2E-02	9.9E-02	1.9E-02	2.4E-01	1.7E-02	2.9E-01	2.7E-02	2.7E-01	6.7E-02
	Pantothenic acid	8.3E-03	1.7E-03	5.9E-03	3.4E-04	2.1E-02	1.3E-03	2.4E-02	5.8E-04	2.2E-02	3.7E-03
	Spermine	5.5E-04	1.9E-04	6.7E-04	9.3E-05	1.1E-03	1.7E-04	1.7E-03	1.6E-04	1.0E-03	8.4E-05
	Taurocholic acid	1.1E-01	1.1E-02	1.3E-01	1.3E-02	3.8E-01	1.1E-01	4.5E-01	6.7E-02	3.7E-01	5.1E-02
	Glycerol 3-phosphate	3.2E-01	4.5E-02	2.1E-01	5.7E-02	6.2E-01	5.1E-03	5.7E-01	1.6E-02	6.3E-01	1.1E-01
	Gluconolactone	6.9E-03	1.6E-03	4.8E-03	3.2E-04	1.0E-02	5.7E-04	9.8E-03	1.2E-03	9.9E-03	1.1E-03
	Glucuronic acid	9.1E-03	4.6E-04	7.0E-03	6.2E-04	1.3E-02	6.3E-04	1.6E-02	5.7E-04	1.3E-02	1.2E-03
	Adenine	1.5E-03	1.6E-04	8.4E-04	1.6E-04	1.6E-03	5.6E-04	2.2E-03	2.2E-04	1.6E-03	2.3E-04
	Sarcosine	4.5E-03	5.8E-04	4.3E-03	2.1E-04	6.7E-03	2.2E-03	8.6E-03	1.5E-03	6.5E-03	1.1E-03
	N-Acetylneuraminic acid	3.3E-03	2.2E-04	3.1E-03	3.6E-04	4.7E-03	1.1E-03	4.8E-03	3.6E-04	4.5E-03	1.0E-03
	UDP-*N*-acetylglucosamine	5.7E-02	4.4E-03	5.8E-02	9.2E-03	8.6E-02	1.6E-02	1.3E-01	4.5E-03	8.4E-02	1.6E-02
	4-Guanidinobutyric acid	8.9E-03	3.9E-04	6.6E-03	6.2E-04	1.4E-01	2.3E-02	1.6E-01	1.3E-03	1.4E-01	9.7E-03
Group 2											
	Cysteinesulfinic acid	3.9E-04	8.1E-05	4.0E-04	5.6E-05	3.5E-04	3.1E-05	2.2E-04	1.6E-05	2.0E-04	1.3E-06
	Uric acid	2.1E-03	1.6E-04	1.9E-03	3.1E-04	1.1E-03	1.1E-04	1.1E-03	1.4E-04	7.1E-04	8.7E-05
	Ribose 5-phosphate	3.1E-03	3.6E-04	2.3E-03	3.7E-04	2.1E-03	4.1E-04	1.4E-03	1.6E-04	1.3E-03	2.8E-04
	Histamine	1.5E-03	1.5E-05	1.3E-03	2.4E-04	4.1E-04	1.1E-04	2.5E-04	4.5E-05	3.2E-04	4.1E-05
	N-Acetylglutamic acid	1.0E-02	8.5E-04	1.0E-02	2.4E-03	7.9E-03	1.2E-03	6.1E-03	4.7E-04	7.2E-03	1.6E-03
	Creatinine	1.0E-02	5.0E-04	6.8E-03	1.6E-04	4.8E-03	7.8E-04	4.7E-03	4.7E-04	3.8E-03	3.9E-04
	6-Phosphogluconic acid	1.1E-02	8.7E-04	9.9E-03	2.6E-03	1.0E-02	1.1E-03	2.3E-03	1.8E-04	1.7E-03	2.1E-04
	Acetoacetic acid	1.5E-02	1.1E-03	1.3E-02	2.3E-03	6.0E-03	9.5E-04	4.2E-03	5.2E-04	3.2E-03	1.5E-03
	Quinolinic acid	1.9E-02	1.9E-03	1.8E-02	6.0E-03	3.3E-03	1.7E-03	4.1E-03	7.4E-04	7.2E-03	1.2E-03
	Succinic acid	3.4E-02	1.4E-03	2.3E-02	4.2E-03	1.7E-02	2.6E-03	1.2E-02	8.6E-04	8.7E-03	1.7E-03
	Citrulline	2.0E-02	1.0E-03	2.7E-02	1.8E-03	7.5E-03	1.8E-03	4.2E-03	9.1E-04	5.0E-03	1.9E-03
	Fructose 6-phosphate	3.2E-02	5.4E-03	1.7E-02	2.7E-03	4.4E-03	3.7E-04	4.0E-03	2.2E-04	3.9E-03	2.3E-04
	Adenosine	7.7E-02	8.9E-03	5.3E-02	5.4E-03	3.5E-02	4.9E-03	3.1E-02	3.6E-03	2.9E-02	5.8E-03
	Met	1.1E-01	4.7E-03	1.2E-01	2.9E-03	8.1E-02	1.4E-02	8.7E-02	3.7E-03	6.7E-02	3.4E-03
	Tyr	1.2E-01	1.4E-02	1.2E-01	4.9E-03	7.9E-02	1.9E-02	7.3E-02	7.3E-03	6.7E-02	1.0E-02
	Glucose 6-phosphate	1.2E-01	1.3E-02	6.4E-02	9.7E-03	1.6E-02	4.1E-03	1.3E-02	3.4E-03	1.2E-02	1.5E-03
	Thr	3.4E-01	1.3E-02	2.9E-01	2.0E-02	2.5E-01	4.0E-02	2.5E-01	8.9E-03	2.0E-01	1.5E-02
	AMP	2.1E-01	6.6E-03	1.6E-01	3.4E-02	1.1E-01	1.2E-02	8.1E-02	8.5E-03	9.2E-02	1.9E-02
	Ile	8.8E-01	4.1E-02	6.8E-01	1.1E-02	5.2E-01	8.5E-02	6.3E-01	2.3E-02	5.0E-01	4.9E-02
	Leu	1.2E+00	6.3E-02	9.8E-01	3.4E-02	7.3E-01	1.3E-01	9.8E-01	4.1E-02	7.7E-01	8.1E-02
Group 3											
	Gln	1.6E-01	1.7E-03	1.9E-01	9.9E-03	1.0E+00	3.4E-02	3.5E+00	1.5E-01	2.6E+00	3.1E-01
	Glu	1.6E+00	4.8E-02	1.7E+00	8.9E-02	4.2E+00	1.4E-01	5.9E+00	1.5E-01	5.8E+00	2.6E-01
	Betaine	4.1E-01	1.8E-02	4.6E-01	1.6E-02	4.1E-01	1.2E-01	1.3E+00	1.2E-02	1.1E+00	5.5E-02
	Asp	1.1E+00	5.4E-02	5.2E-01	5.4E-02	1.7E+00	2.9E-02	2.1E+00	5.5E-02	2.1E+00	1.2E-01
	Ergothioneine	1.1E-01	5.6E-03	9.7E-02	5.0E-03	1.2E-01	1.5E-02	3.3E-01	4.0E-03	3.0E-01	1.6E-02
	O-Acetylcarnitine	1.5E-01	5.2E-03	1.2E-01	4.8E-03	2.2E-01	1.8E-02	3.5E-01	9.7E-03	3.5E-01	2.3E-02
	Gly	2.3E-01	1.6E-02	1.7E-01	1.0E-03	3.7E-01	2.1E-02	6.5E-01	1.0E-02	4.9E-01	4.8E-02
	Carnitine	1.7E-02	5.9E-04	2.0E-02	1.2E-03	3.4E-02	4.2E-03	6.1E-02	2.3E-03	7.1E-02	5.0E-03
	β-Ala	4.5E-03	2.5E-04	3.3E-03	1.6E-04	8.2E-03	1.3E-03	2.1E-02	1.2E-03	2.1E-02	1.5E-03
	Taurine	2.3E-03	3.3E-04	1.8E-03	2.2E-04	6.5E-03	3.9E-04	1.4E-02	1.1E-03	1.5E-02	1.0E-03
	Saccharopine	6.5E-03	2.1E-04	6.5E-03	2.5E-04	9.2E-03	1.4E-03	1.7E-02	5.0E-04	1.3E-02	6.2E-04
	N-Acetylornithine	9.4E-04	1.8E-04	1.4E-03	5.3E-05	1.5E-03	3.9E-04	5.1E-03	5.4E-04	4.3E-03	3.3E-04
	Kynurenic acid	2.4E-03	1.5E-04	1.8E-03	2.0E-04	2.1E-03	3.6E-04	4.1E-03	1.8E-04	3.7E-03	3.7E-04

Supplementary Table 1 J. Ishikawa *et al*.

Compound name	Relative Area 4°C Mean	4°C S.D.	10°C Mean	10°C S.D.	22°C Mean	22°C S.D.	33°C Mean	33°C S.D.	37°C Mean	37°C S.D.
Group 4										
Inosine	8.1E-03	1.9E-03	8.7E-03	1.7E-03	6.5E-03	1.7E-03	5.8E-03	6.7E-04	5.1E-03	7.7E-04
Citramalic acid	5.6E-03	5.4E-04	5.0E-03	1.1E-03	8.4E-03	5.8E-04	6.2E-03	5.4E-04	6.3E-03	1.5E-03
CDP	6.7E-03	9.1E-04	5.7E-03	1.6E-03	4.4E-03	2.2E-04	4.4E-03	1.8E-04	3.8E-03	2.7E-04
ADP	3.7E-01	3.4E-02	3.4E-01	7.7E-02	3.1E-01	3.2E-02	2.4E-01	5.1E-03	2.6E-01	6.0E-02
Ethanolamine phosphate	3.0E-03	1.6E-04	2.7E-03	8.9E-05	2.9E-03	5.3E-04	3.7E-03	3.8E-04	4.4E-03	2.1E-04
IMP	5.9E-03	1.0E-03	2.3E-03	4.0E-04	1.0E-03	2.4E-04	7.2E-04	4.5E-05	8.3E-04	1.4E-04
Ribulose 5-phosphate	1.2E-02	1.4E-03	6.6E-03	1.4E-03	5.3E-03	8.9E-04	4.3E-03	3.2E-04	3.9E-03	9.2E-04
Sedoheptulose 7-phosphate	1.8E-02	3.2E-03	1.3E-02	1.7E-03	1.6E-02	2.4E-03	5.6E-03	9.4E-05	3.0E-03	4.9E-04
Phosphorylcholine	1.1E-01	4.8E-03	9.1E-02	7.1E-03	6.0E-02	9.1E-03	3.4E-01	8.5E-03	4.1E-01	1.4E-02
GMP	1.1E-02	8.1E-04	8.2E-03	1.5E-03	8.6E-03	1.7E-03	5.9E-03	5.3E-04	5.8E-03	4.4E-04
CoA_divalent	1.6E-02	3.9E-03	2.2E-02	4.1E-03	1.1E-02	2.3E-03	1.2E-02	2.1E-03	1.4E-02	4.2E-04
Ethanolamine	4.4E-03	2.3E-04	5.6E-03	4.9E-04	4.3E-03	1.1E-03	9.5E-03	1.9E-04	9.9E-03	1.5E-03
Pro	7.9E-02	8.4E-03	5.8E-02	2.5E-03	7.7E-02	1.5E-02	1.0E-01	5.6E-03	8.3E-02	7.5E-03
Val	9.0E-01	3.4E-02	7.6E-01	2.5E-02	6.6E-01	8.5E-02	8.1E-01	1.7E-02	6.4E-01	5.3E-02
CDP-choline	3.6E-04	1.1E-04	3.2E-04	2.0E-05	3.9E-04	2.2E-04	8.5E-04	1.8E-04	1.9E-03	4.9E-04
Choline	1.2E-01	2.1E-03	1.3E-01	3.6E-03	5.4E-02	1.2E-02	4.3E-01	4.4E-03	5.1E-01	2.2E-02
Cytidine	2.5E-02	2.5E-03	2.5E-02	2.2E-03	4.1E-02	5.1E-03	3.4E-02	2.2E-03	2.4E-02	1.2E-03
Pyruvic acid	4.3E-02	9.7E-03	2.2E-02	2.5E-03	2.1E-02	3.8E-03	1.8E-02	2.9E-03	1.1E-02	2.6E-03
3-Hydroxybutyric acid	2.5E-01	1.4E-02	1.4E-01	1.4E-02	2.9E-01	2.2E-02	2.9E-01	1.4E-02	2.4E-01	5.3E-02
Creatine	1.3E-02	2.4E-04	8.9E-03	3.5E-04	7.4E-03	9.4E-04	9.2E-03	5.3E-04	7.9E-03	1.1E-03
UDP	1.9E-02	2.8E-03	2.2E-02	4.4E-03	1.7E-02	1.1E-03	1.6E-02	9.3E-04	1.5E-02	1.5E-03
Guanosine	6.4E-03	7.7E-04	6.4E-03	3.9E-04	1.1E-02	1.6E-03	8.1E-03	5.7E-04	6.2E-03	6.9E-04
ATP	8.7E-01	1.4E-01	9.6E-01	1.4E-01	1.2E+00	1.2E-01	1.2E+00	9.7E-02	9.9E-01	2.4E-01
Phe	1.7E-01	1.9E-02	1.8E-01	5.4E-03	1.1E-01	2.3E-02	1.8E-01	1.2E-02	1.4E-01	1.7E-02
Arg	9.4E-04	3.8E-04	1.1E-03	1.0E-03	1.1E-03	1.1E-04	2.2E-03	6.3E-05	2.5E-03	6.6E-04
CMP	4.4E-03	5.3E-04	2.6E-03	5.9E-04	2.4E-03	2.7E-04	2.3E-03	1.6E-04	2.1E-03	6.0E-05
Kynurenine	1.8E-03	3.5E-04	1.3E-03	7.7E-05	1.1E-03	3.9E-04	1.1E-03	1.4E-04	1.3E-03	2.3E-04
Ornithine	1.3E-01	9.3E-03	1.2E-01	7.3E-03	1.6E-01	2.5E-02	1.6E-01	1.1E-02	1.2E-01	1.5E-02
2-Aminoadipic acid	8.0E-02	3.7E-03	3.9E-02	5.1E-03	1.6E-02	2.9E-03	4.5E-02	3.7E-03	4.5E-02	2.6E-03
Glucose 1-phosphate	1.1E-01	8.5E-03	4.8E-02	6.3E-03	6.6E-02	6.4E-03	3.5E-02	1.9E-03	3.2E-02	3.3E-03
Urea	1.2E-01	3.4E-02	1.2E-01	1.8E-02	1.6E-01	3.4E-02	1.4E-01	1.2E-02	1.7E-01	6.1E-02
S-Lactoylglutathione	1.7E-03	9.4E-05	2.1E-03	2.5E-04	3.1E-03	1.3E-03	2.0E-03	2.4E-04	2.4E-03	2.9E-04
Fumaric acid	4.3E-02	3.4E-03	2.5E-02	6.7E-03	3.8E-02	5.6E-03	4.8E-02	5.2E-03	4.8E-02	9.5E-03
Malic acid	4.0E-01	3.7E-02	2.4E-01	7.0E-02	3.6E-01	6.4E-02	4.7E-01	4.4E-02	4.3E-01	8.2E-02
Glycerophosphocholine	5.2E-01	3.8E-02	5.0E-01	3.4E-02	2.4E-01	7.1E-02	8.6E-01	2.0E-02	1.5E+00	1.2E-01
Asn	2.9E-02	1.4E-03	2.7E-02	4.3E-04	2.4E-02	2.3E-03	4.2E-02	2.2E-03	3.3E-02	2.1E-03
cis-Aconitic acid	2.3E-02	2.6E-04	2.2E-02	6.0E-03	3.1E-02	3.6E-03	9.0E-03	5.8E-04	7.7E-03	1.9E-03
Acetyl CoA_divalent	8.5E-04	1.2E-04	5.3E-04	1.7E-04	7.6E-04	1.4E-04	3.8E-04	9.5E-05	4.0E-04	1.0E-04
Trp	1.8E-02	1.6E-03	2.7E-02	1.1E-03	2.0E-02	2.6E-03	2.2E-02	1.8E-03	1.4E-02	1.6E-03
Phosphoenolpyruvic acid	1.3E-02	3.1E-03	7.8E-03	1.2E-03	1.2E-02	1.6E-03	1.5E-02	1.3E-03	1.1E-02	4.3E-03
Argininosuccinic acid	2.4E-03	5.9E-05	2.8E-03	3.4E-05	2.7E-03	5.3E-04	2.4E-03	3.3E-04	1.8E-03	2.0E-04
Erythrose 4-phosphate	7.1E-03	7.6E-05	5.3E-03	7.0E-04	6.8E-03	1.1E-03	5.2E-03	6.2E-04	4.1E-03	8.6E-04
Glutathione (GSH)	2.7E+00	9.7E-02	2.9E+00	1.1E-01	2.4E+00	5.9E-01	3.9E+00	2.7E-01	3.1E+00	3.4E-01
Nicotinamide	7.4E-03	5.0E-04	4.4E-03	3.3E-04	6.0E-03	7.2E-04	4.7E-03	5.5E-04	4.1E-03	2.6E-04
2,3-Diphosphoglyceric acid	6.8E-03	9.4E-04	7.5E-03	1.9E-03	2.0E-02	2.4E-03	7.4E-03	8.3E-04	3.2E-03	1.0E-03
2-Oxoglutaric acid	2.6E-02	1.8E-03	5.5E-02	1.2E-02	3.1E-02	1.8E-03	3.5E-02	2.8E-03	2.8E-02	4.6E-03
NAD^+	1.5E-01	1.5E-02	1.5E-01	1.4E-02	1.8E-01	7.1E-02	1.4E-01	2.3E-02	1.7E-01	5.9E-02
N^6,N^6,N^6-Trimethyllysine	1.3E-02	3.3E-04	7.7E-03	3.0E-04	2.7E-03	3.7E-04	1.7E-02	9.3E-04	2.1E-02	2.2E-03
CTP	2.1E-02	1.4E-03	1.9E-02	5.1E-03	1.9E-02	9.2E-04	2.0E-02	7.2E-04	1.7E-02	5.4E-03
3-Methylhistidine	3.4E-03	2.6E-04	2.9E-03	2.5E-04	2.9E-03	5.1E-04	3.3E-03	1.8E-04	2.7E-03	2.6E-04
CMP-*N*-acetylneuraminate	1.4E-02	3.5E-04	1.4E-02	3.2E-03	1.7E-02	3.9E-03	9.7E-03	5.5E-04	1.2E-02	3.0E-03
2-Phosphoglyceric acid	3.9E-03	1.2E-03	2.6E-03	3.0E-04	3.2E-03	2.2E-04	4.5E-03	3.3E-04	3.5E-03	1.5E-03
γ-Butyrobetaine	4.6E-03	2.2E-04	3.7E-03	4.0E-04	7.2E-04	1.7E-04	3.7E-03	3.8E-04	5.9E-03	6.9E-04
UTP	7.1E-02	4.7E-03	7.7E-02	1.4E-02	7.4E-02	7.7E-03	9.1E-02	9.2E-03	7.5E-02	2.4E-02
$NADP^+$	1.2E-01	1.1E-02	1.2E-01	2.7E-02	1.3E-01	3.6E-02	1.4E-01	4.9E-03	1.2E-01	3.8E-02
Fructose 1,6-diphosphate	6.9E-03	6.0E-04	6.6E-03	1.6E-03	9.8E-03	1.3E-03	7.0E-03	3.9E-04	5.5E-03	1.9E-03
UMP	1.9E-02	2.6E-03	1.5E-02	3.3E-03	2.4E-02	8.8E-03	1.7E-02	3.9E-03	1.5E-02	1.1E-03
Hydroxyproline	4.3E-03	6.2E-05	2.5E-03	3.4E-04	3.2E-03	2.7E-04	4.3E-03	3.5E-04	3.9E-03	1.1E-04
S-Adenosylhomocysteine	1.8E-03	2.3E-04	1.7E-03	2.3E-04	2.6E-03	7.6E-04	1.8E-03	3.0E-04	1.3E-03	2.5E-04
3-Phosphoglyceric acid	3.1E-02	6.2E-03	1.8E-02	2.8E-03	2.4E-02	2.2E-03	3.3E-02	2.0E-03	2.4E-02	1.1E-02
ADP-ribose	9.7E-04	1.9E-04	8.0E-04	8.0E-05	1.2E-03	1.7E-04	9.1E-04	7.8E-05	7.6E-04	6.4E-05
Glycerol	7.3E-01	2.1E-01	6.5E-01	5.1E-02	5.5E-01	2.0E-01	5.8E-01	8.7E-02	7.8E-01	3.0E-01
Glyceric acid	4.2E-03	3.9E-04	2.4E-03	3.0E-04	3.7E-03	7.5E-04	4.1E-03	3.4E-04	2.9E-03	8.5E-04
2-Hydroxybutyric acid	9.8E-03	6.2E-04	6.4E-03	4.9E-04	9.8E-03	4.5E-04	8.7E-03	2.8E-04	7.0E-03	1.7E-03
Hypotaurine	5.5E-03	5.7E-04	4.9E-03	3.8E-04	4.9E-03	1.3E-03	6.1E-03	2.9E-04	5.1E-03	2.7E-04
Cys	6.9E-03	4.4E-03	1.2E-02	1.7E-03	6.0E-03	5.9E-03	7.8E-03	7.4E-03	1.2E-02	7.2E-03
Lys	1.0E+00	5.9E-02	1.0E+00	7.1E-02	1.4E+00	1.6E-01	9.5E-01	4.4E-02	6.2E-01	2.6E-02
Cystathionine	3.8E-03	1.9E-04	3.6E-03	2.6E-04	5.4E-03	1.5E-03	4.1E-03	4.2E-04	2.0E-03	8.9E-05
GDP	2.1E-02	2.1E-03	2.1E-02	4.5E-03	2.5E-02	8.1E-04	1.9E-02	1.9E-03	2.0E-02	2.8E-03
Putrescine	1.9E-02	1.4E-03	1.9E-02	1.9E-03	2.8E-02	2.7E-03	2.0E-02	1.4E-03	9.8E-03	1.7E-03
Citric acid	5.1E-01	6.2E-02	4.6E-01	1.1E-01	9.5E-01	3.8E-02	2.3E-01	1.8E-02	2.0E-01	4.0E-02
Lactic acid	4.1E-01	3.1E-02	2.4E-01	2.2E-02	3.7E-01	3.4E-02	3.1E-01	8.2E-03	2.7E-01	4.3E-02
NMN	1.4E-02	3.1E-03	1.6E-02	3.1E-03	2.1E-02	9.4E-03	1.3E-02	3.7E-03	1.3E-02	6.0E-03

SUPPLEMENTARY FIGURE LEGENDS

Supplementary Figure 1. Optimisation of organ perfusion culture system.

a, Photographs and angiographic images by micro-CT of the perfusion area in natural and cultured liver through the liver placement including *in vivo* (*left column*), flat putting on culture dish (*centre column*) and hanging with costal arch floated in the organ chamber (*right column*). Scale bars, 1 cm.

b, Assessments of culture solution temperature (*upper left*), concentration of dissolved oxygen (*upper right*) and pH (*lower*) during normothermic liver perfusion culture with/without erythrocytes. These data represent the erythrocyte concentration of 0.5×10^{11} cells/L (green), 2.0×10^{11} cells/L (orange), with 5.0×10^{11} cells/L (red) and no erythrocytes (blue).

c, Histological analysis of sinusoidal structure in cultured liver after 48-hrs perfusion culture at hypothermic condition (22°C) with erythrocyte concentrations of 0.5×10^{11} cells/L (*left*), 2.0×10^{11} cells/L (*right*). Scale bars, 100 μm.

Supplementary Figure 2. Analysis of cell behaviour and intracellular metabolism due to culture temperature.

a, Phase-contrast images of Huh 7 cells under various culture temperatures of 4, 10, 22, 33 and 37°C. Representative data for the culture periods for 24-hrs (*top*) and 96-hrs (*bottom*) are shown. Scale bars, 100 μm.

b, Heat map image of clustering analysis that compares the intracellular metabolites of hepatocytes under various culture temperatures. The heat map represents the z-value of concentration in each metabolite.

Supplementary Figure 3. Analysis of intracellular metabolism due to culture temperature with a metabolic pathway map.

The metabolic pathway maps of cultured hepatocytes under various temperatures of 4, 10, 22, 33 and 37°C. The high-accumulated metabolites in the pathway map are represented by the coloured dots in the lower temperature condition (4, 10°C; blue), the hypothermic and body temperature condition (22, 33, 37°C; red) and the dependence with temperature increase (green).

Supplementary Figure 4. Assessment of resuscitation in DCD liver by the hypothermic perfusion culture.

a, Assessment of ALT activity in DCD liver during *ex vivo* liver perfusion culture with/without erythrocyte at 22°C for 48-hrs. These data represent by addition of 5.0×10^{11} cells/L erythrocyte (red) and no erythrocytes (white).

b, Evaluation of albumin synthesis in DCD liver during *ex vivo* liver perfusion culture with/without erythrocyte at 22°C for 48-hrs. These data represent the addition of 5.0×10^{11} cells/L erythrocyte (red) and no erythrocytes (white).

c, Histological analysis of natural liver (*left*), ischaemic liver after 90-min cardiac arrest (*centre left*) and cultured ischaemic liver after 48-hrs perfusion culture at 22°C with/without erythrocytes (*centre right and right*). Higher magnification images are shown in the boxed area (*bottom*). Scale bars, 100 μm.

d, Histological analysis of natural liver (*left*) and engrafted donor liver at 7 days after transplantation including a cold preservative liver with UW solution (*centre left*), a cultured liver without erythrocytes for 100-min (*centre right*) and a cultured liver with

erythrocytes for 100-min (*right*). Higher magnification images of the sinusoidal structure are shown in the boxed area (*middle and bottom*). Scale bars, 100 μm.

SUPPLEMENTARY TABLE LEGENDS

Supplementary Table 1. Accumulated value of metabolites in primary hepatocyte depending on culture temperature.

The major metabolites, which are depicted in the metabolism pathway map, are detected by the metabolome analysis of rat primary hepatocyte culture for various culture temperatures. Categorisation of specific metabolites was performed using statistical analysis by employing a threshold value of 1.4-fold change or more; the metabolites were divided into four groups. Group 1 represents the higher metabolite accumulation of the 22, 33 and 37°C culture conditions compared with the 4 and 10°C culture, Group 2 represents the higher metabolite accumulation of 4 and 10°C culture conditions compared with 22, 33 and 37°C culture, Group 3 represents the accumulated metabolites dependent on temperature increase, and Group 4 represents the metabolites for which the relative concentrations were not significantly detected in each culture temperature. These data are presented along with the relative values calculated by

CE-TOFMS optional software (PeakStat ver.3.18; HMT, Tsuruoka, Japan). The metabolites that exhibited a “Not detected” value were excluded in this analysis.
